# Supplementary material for: Genetic Entanglement Enables Ultrastable Biocontainment in the Mammalian Gut
Source: ACS Synth Biol. 2025 Sep 7;14(9):3696–708. doi: 10.1021/acssynbio.5c00412 (PMC12455656; doi:10.1021/acssynbio.5c00412)
Supplement: Supplementary file 1 [file sb5c00412_si_001.pdf]

**Supplemental Data for:**

# Genetic entanglement enables ultra-stable biocontainment in the mammalian gut

Gary W. Foo[1], Aathavan S. Uruthuripathy[1], Claire Q. Zhang[1], Izabela Z. Batko[2], David E. Heinrichs[2] and David R. Edgell[1*]

[1]Department of Biochemistry, Schulich School of Medicine and Dentistry, London, Ontario N6A 5C1, Canada
[2]Department of Microbiology and Immunology, Schulich School of Medicine and Dentistry, London, Ontario N6A5C1, Canada
* corresponding author: dedgell@uwo.ca

**This document includes:**
Figs. S1-S7

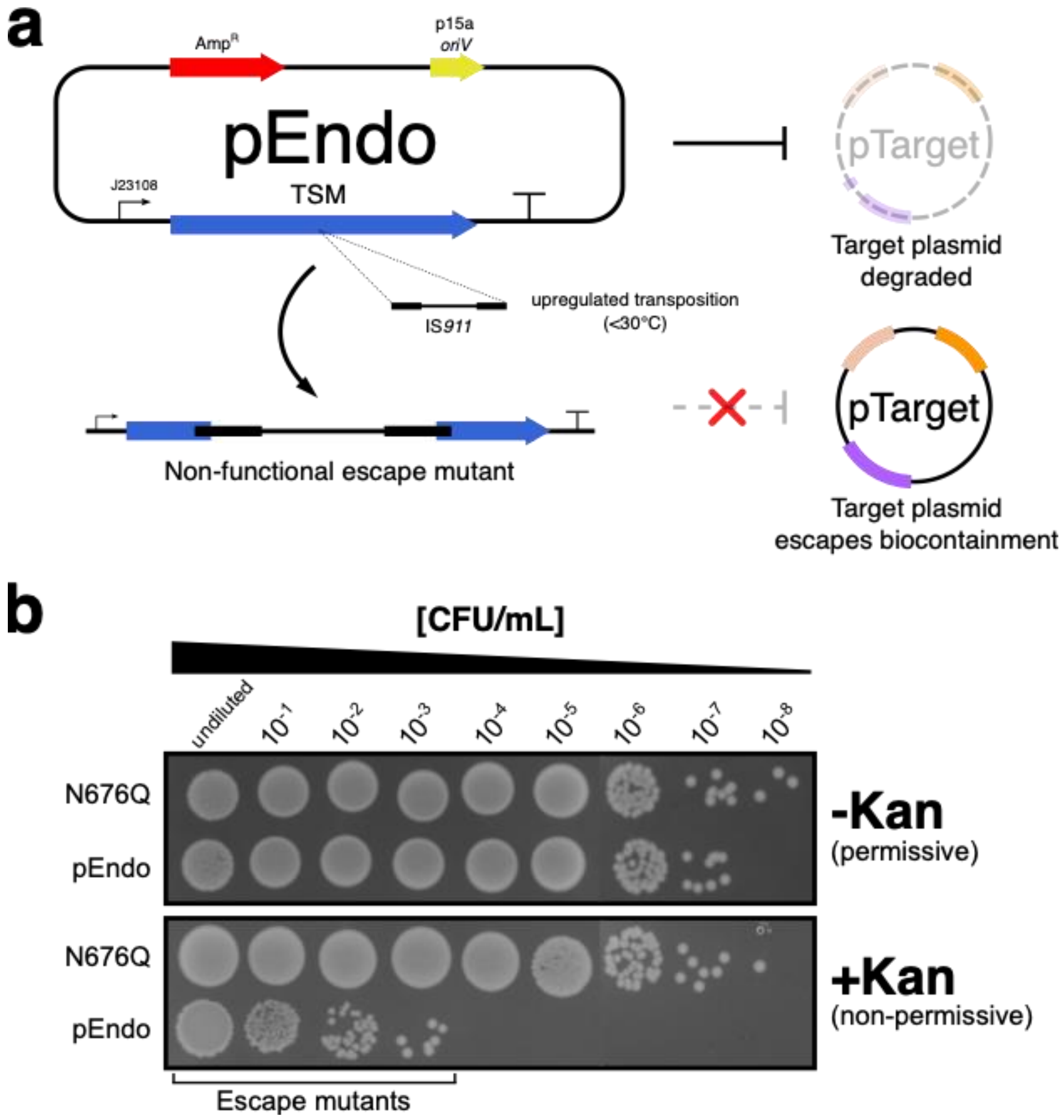


**Fig S1. Escape mechanism and frequency for the original I-OnuI TSM. (A)** Primary mechanism for circuit escape with the original TSMs. **(B)** I-OnuI TSM activity in *E. coli* on permissive and non-permissive LB plates, incubated at 18°C. Growth observed with the pEndo TSM on non-permissive media are considered escape mutants. The N676Q negative control is incapable of intein-splicing.

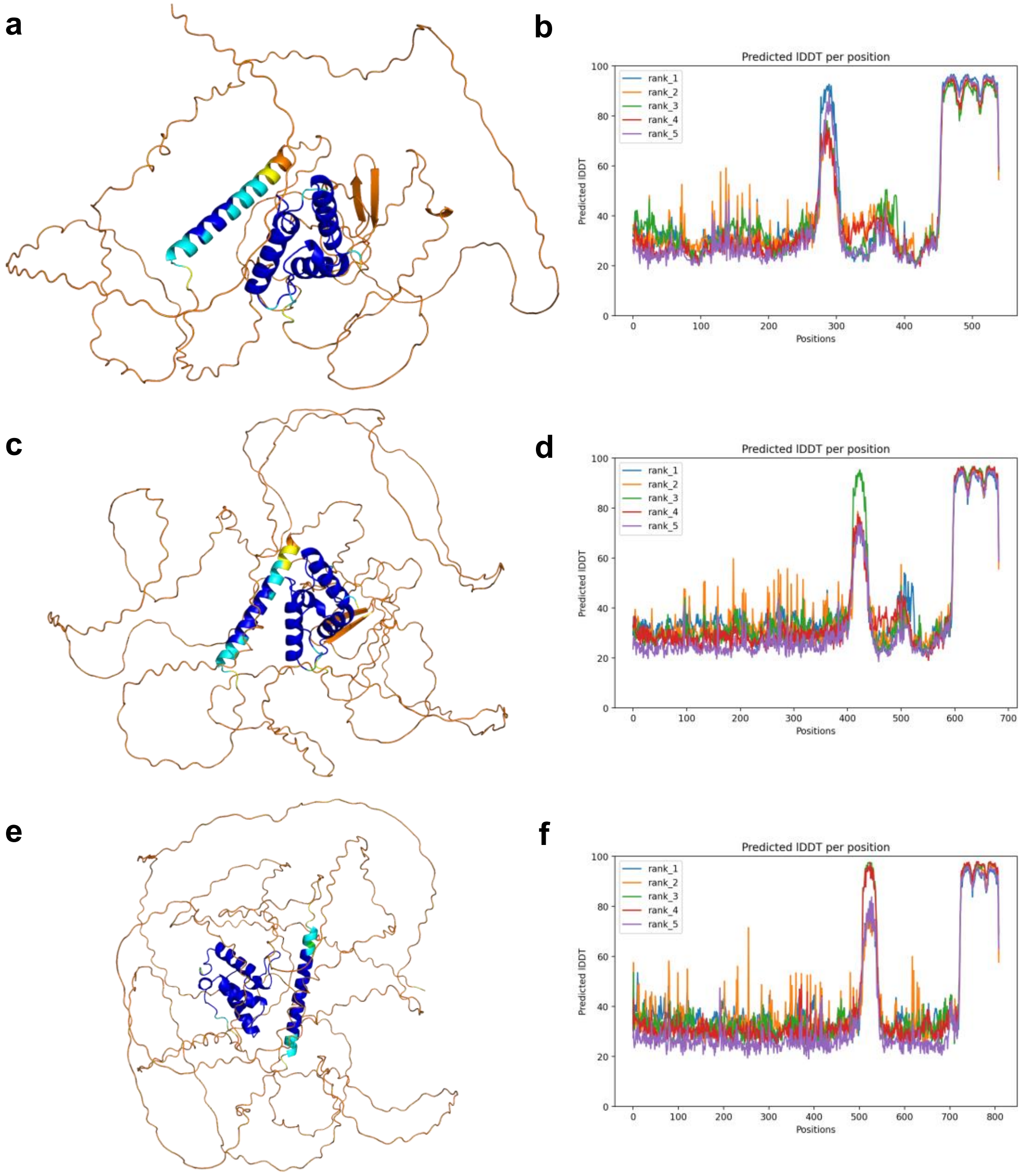


**Fig S2. AlphaFold2 predicted structures for STALEMATE constructs using Im9.** **(A,C,E)** Predicted structures for Onu-933 (A), Onu-533 (C), and Pan-126 (E) coloured by pLDDT. **(B,D,F)** pLDDT plots for Onu-933 (B), Onu-533 (D), and Pan-126 (F).

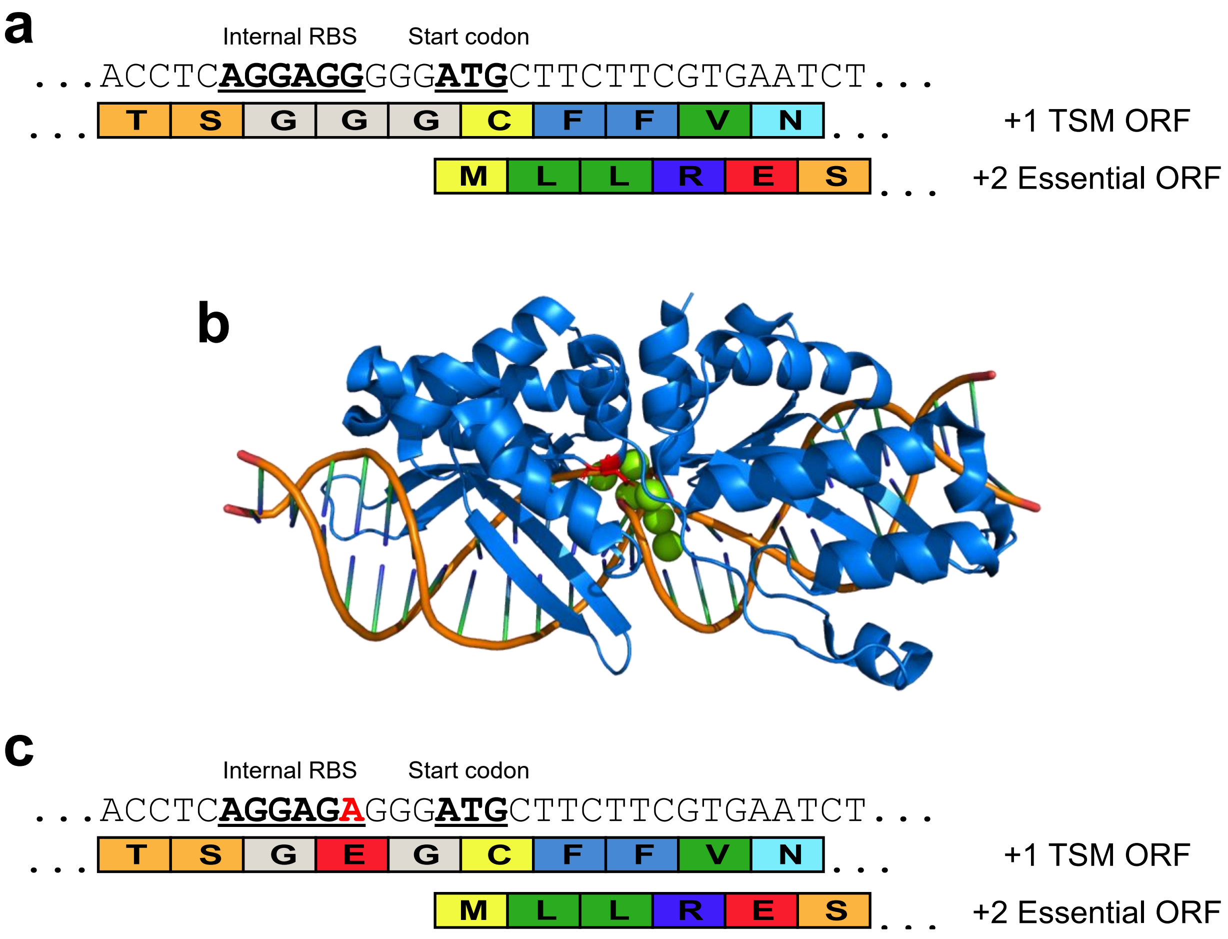


**Fig S3. Sequence of Onu-533 before and after restoring activity. (A)** The creation of the cognate AGGAGG ribosome binding site (RBS) in the +2 reading frame created the E180G catalytically inactive I-OnuI variant. **(B)** Crystal structure of I-OnuI (PDB:6BDA) in blue bound to its cognate target site. In red is the location of E180, one of the two residues coordinating the divalent metal ion cofactors shown in green. **(C)** Sequence of the corrected version of Onu-533, using an alternative RBS (AGGAGA) to restore E180. Shown in red is the nucleotide altered to make the change

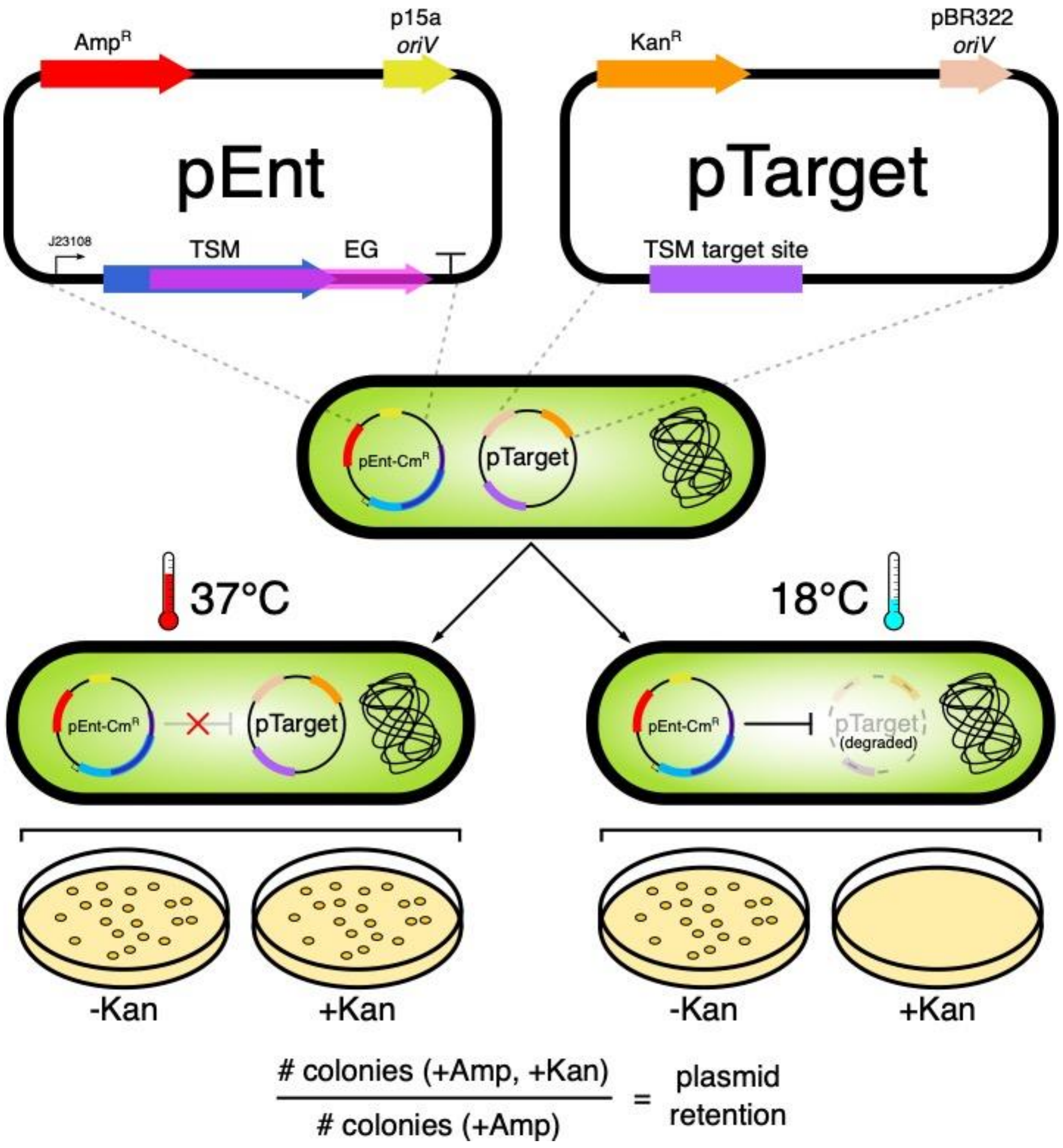


**Fig S4. Two-plasmid cleavage assay.** Plasmid maps for pEnt and pTarget used in this study. *oriV*, plasmid origin of replication; $Amp^R$, ampicillin resistance gene; $Kan^R$, kanamycin resistance gene; TSM, thermoregulated meganuclease; EG, essential gene; J23108, one of the Anderson promoters. The TSM target site is only cleaved when TSMs are reconstituted at 18°C and is measurable by the loss of kanamycin resistance on selective media.

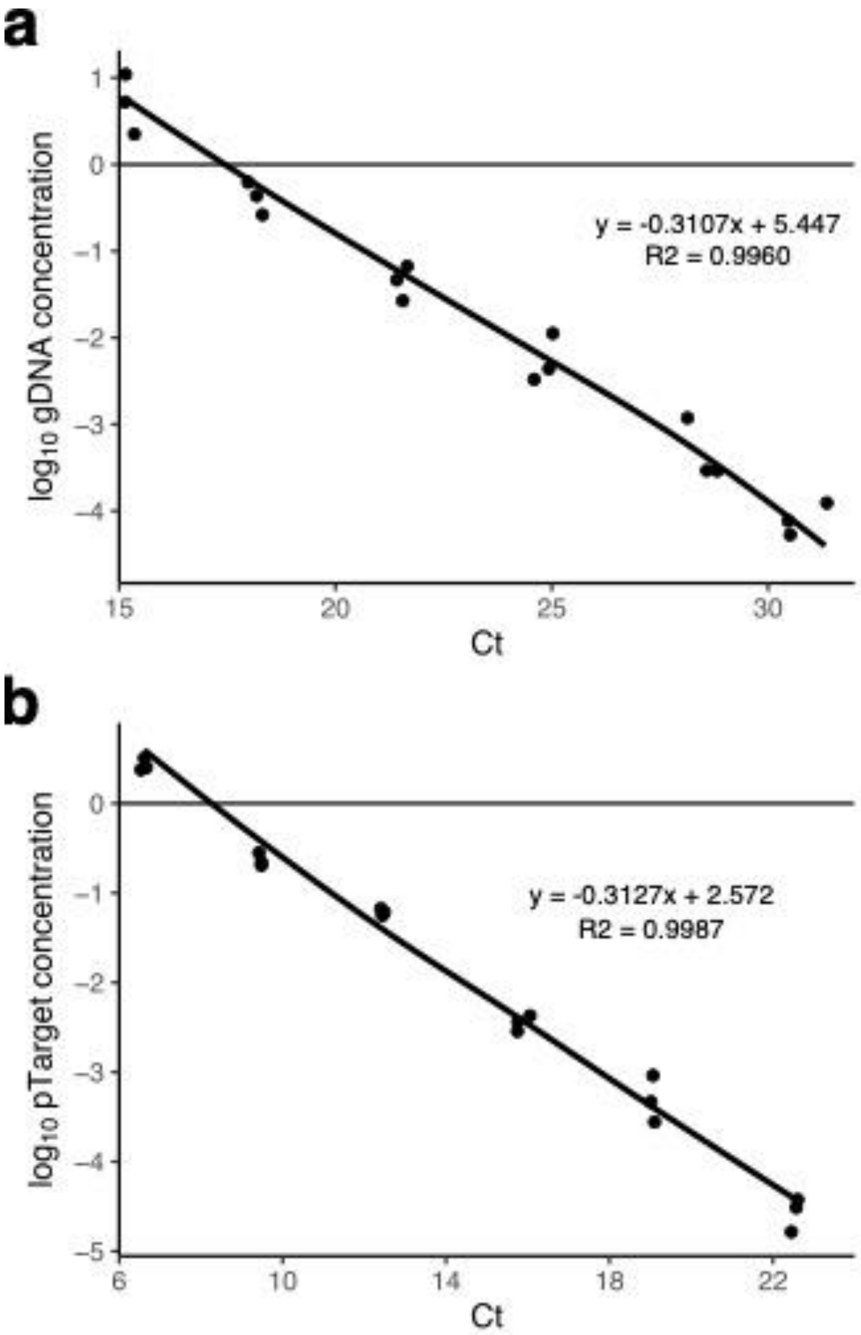


**Fig S5. qPCR standard curves for *E. coli* Nissle 1917 gDNA (A) and pTarget (B).** qPCR was performed with SYBR Select Master Mix (Applied Biosystems) on the Viia7 (ThermoFisher Scientific). The primer pair DE-7269 and DE-7270 were used against a 150 bp amplicon on the pTarget kanamycin resistance gene. The primer pair DE-7271 and DE-7272 targeted the *CspA* gene on the *E. coli* Nissle 1917 chromosome, also amplifying a 150 bp amplicon.

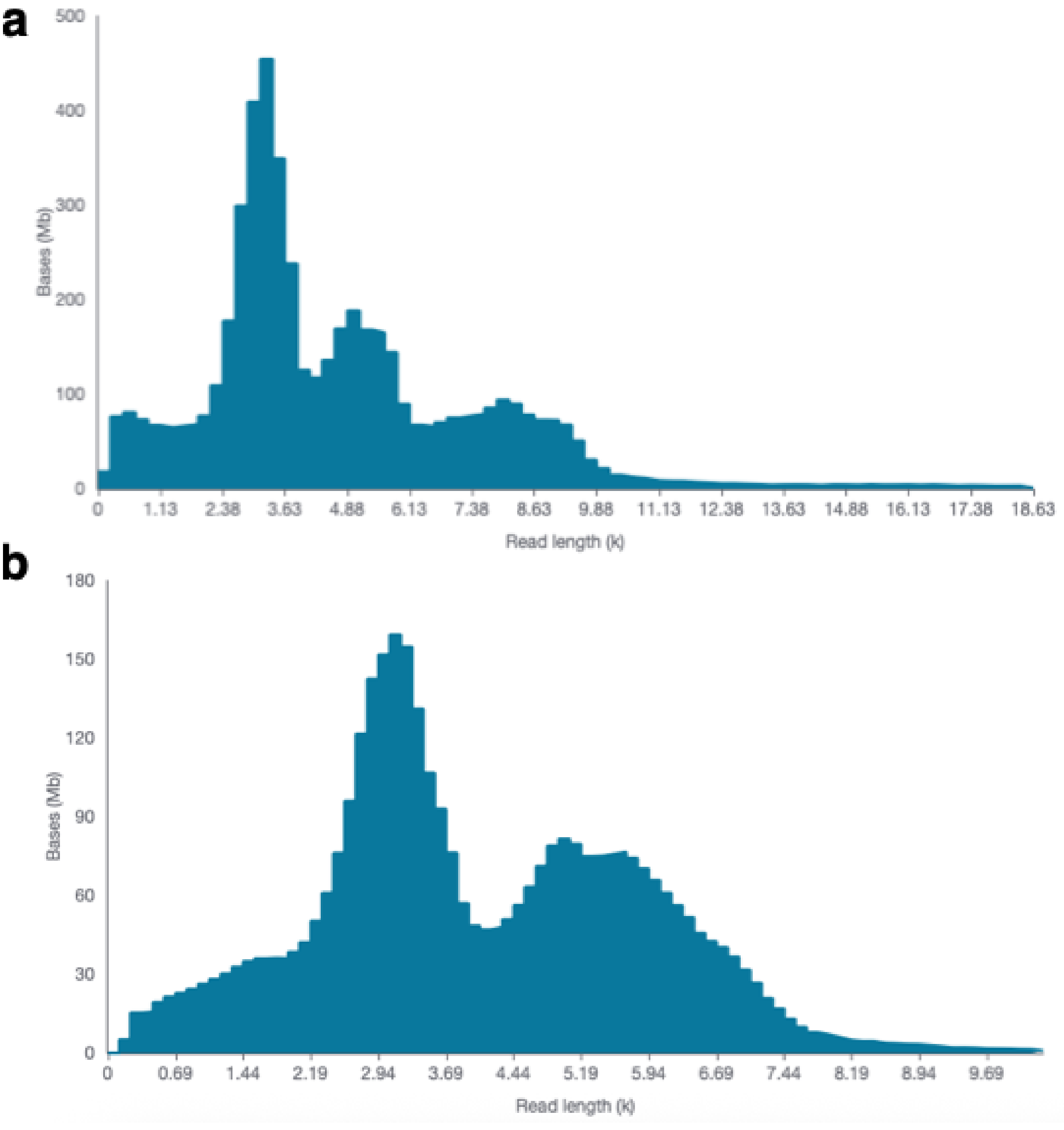


**Fig S6. Representative read length histograms from escape mutant sequencing.** Both plots potentially carry up to four plasmid species in the pool, two designed in this study (pEndo/pEnt and pTarget (~3.7 kb)), and two cryptic plasmids found in *E. coli* Nissle 1917 (pMut1 (~3.2 kb) and pMut2 (~5.5 kb)). **(A)** Histograms for pEnt-533/pEnt-933 (~7.6 kb normally, ~8.9 kb with IS*911*). **(B)** Histogram for pEndo (~5.2 kb, ~6.7 kb with IS*911*).

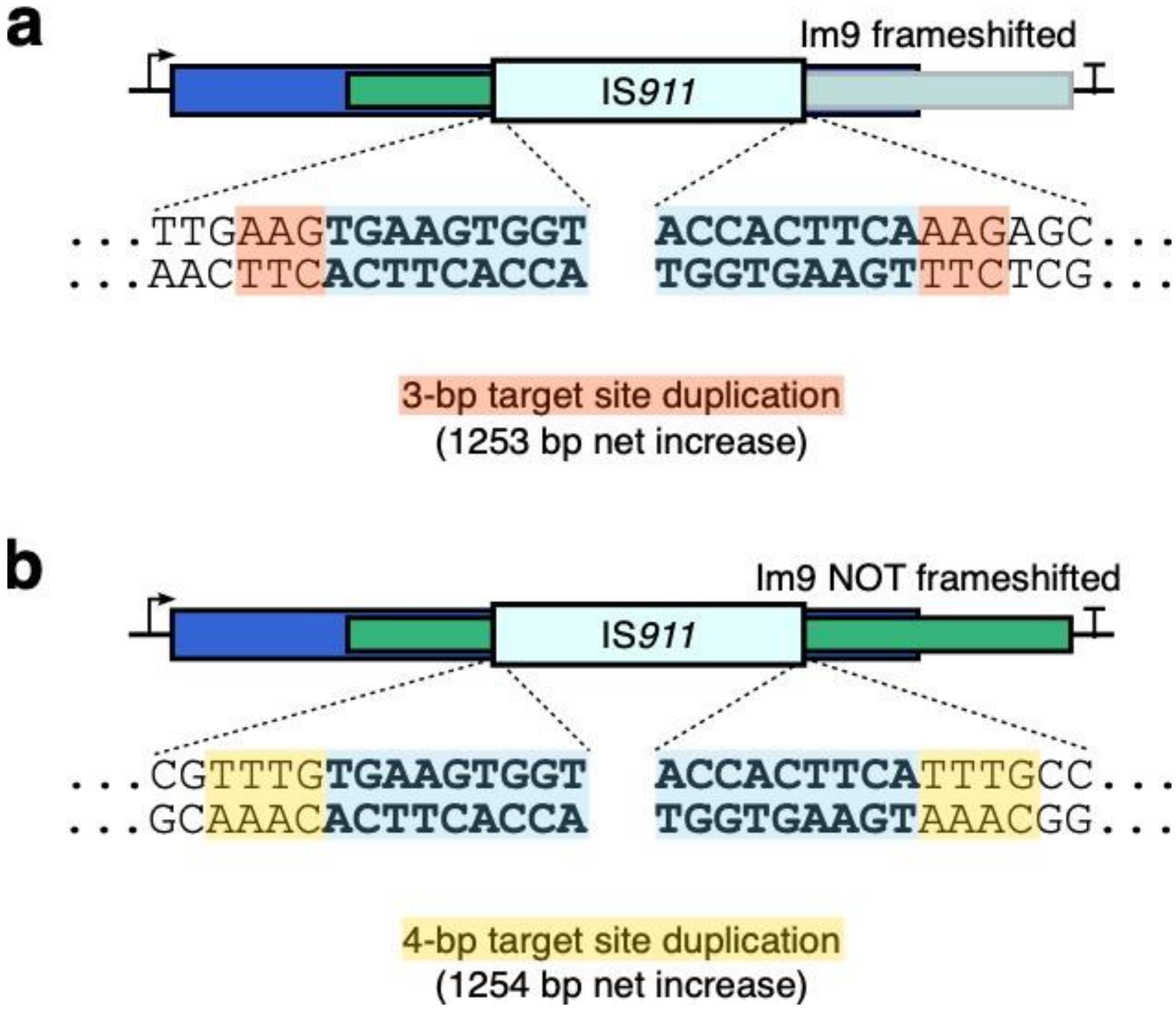


**Fig S7. Alternative IS*911* target site duplication as a mechanism for escape. (A)** Shown is the canonical 3-bp target site duplication that occurs during IS*911* transposition. IS*911* is 1250 bp long, and therefore results in a net 1253 bp increase, which causes a frameshift no matter the reading frame. **(B)** More rarely, a 4-bp target site duplication can occur and can result in a 1254 bp net increase. Depending on the orientation, this can result in a frameshift in one, but not the other reading frames.
