# Supplementary material for: Genetic Entanglement Enables Ultrastable Biocontainment in the Mammalian Gut
Source: ACS Synth Biol. 2025 Sep 7;14(9):3696–708. doi: 10.1021/acssynbio.5c00412 (PMC12455656; doi:10.1021/acssynbio.5c00412)
Supplement: Supplementary file 8 [file sb5c00412_si_008.pdf]

```
LOCUS       pTarget      3721 bp ds-DNA     circular     09-AUG-2023
DEFINITION  .
FEATURES             Location/Qualifiers
     misc_feature    898..919
                     /label="I-OnuI Target Site"
                     /ApEinfo_revcolor="#d59687"
                     /ApEinfo_fwdcolor="#d59687"
     misc_feature    1092..1943
                     /label="pBR332deltaROM"
                     /ApEinfo_revcolor="#b7e6d7"
                     /ApEinfo_fwdcolor="#b7e6d7"
     misc_feature    2067..2195
                     /label="Double Terminator"
                     /ApEinfo_revcolor="#f8d3a9"
                     /ApEinfo_fwdcolor="#f8d3a9"
     misc_feature    2197..3573
                     /label="Cen6-ArsH4-His3"
                     /ApEinfo_revcolor="#b1ff67"
                     /ApEinfo_fwdcolor="#b1ff67"
     misc_feature    3574..3697
                     /label="KanR promoter"
                     /ApEinfo_revcolor="#b7e6d7"
                     /ApEinfo_fwdcolor="#b7e6d7"
     misc_feature    3698..792
                     /label="KanR"
                     /ApEinfo_revcolor="#b1ff67"
                     /ApEinfo_fwdcolor="#b1ff67"
     CDS             3698..792
                     /ApEinfo_revcolor="#84b0dc"
                     /ApEinfo_fwdcolor="#84b0dc"

/translation="MSHIQRETSCSRPRLNSNMDADLYGYKWARDNVGQSGATIYRLYGKPDAPELFLKHGKG
SVANDVTDEMVRLNWLTEFMPLPTIKHFIRTPDDAWLLTTAIPGKTAFQVLEEYPDSGENIVDALAVFLRRLH
SIPVCNCPFNSDRVFRLAQAQSRMNNGLVDASDFDDERNGWPVEQVWKEMHKLLPFSPDSVVTHGDFSLDNLI
FDEGKLIGCIDVGRVGIADRYQDLAILWNCLGEFSPSLQKRLFQKYGIDNPDMNKLQFHLMLDEFF*"
ORIGIN
        1 tcttgctcga ggccgcgatt aaattccaac atggatgctg atttatatgg
gtataaatgg
       61 gctcgcgata atgtcgggca atcaggtgcg acaatctatc gattgtatgg
gaagcccgat
      121 gcgccagagt tgtttctgaa acatggcaaa ggtagcgttg ccaatgatgt
tacagatgag
      181 atggtcagac taaactggct gacggaattt atgcctcttc cgaccatcaa
gcattttatc
      241 cgtactcctg atgatgcatg gttactcacc actgcgatcc ccgggaaaac
agcattccag
      301 gtattagaag aatatcctga ttcaggtgaa aatattgttg atgcgctggc
agtgttcctg
      361 cgccggttgc attcgattcc tgtttgtaat tgtcctttta acagcgatcg
cgtatttcgt
      421 ctcgctcagg cgcaatcacg aatgaataac ggtttggttg atgcgagtga
ttttgatgac
      481 gagcgtaatg gctggcctgt tgaacaagtc tggaaagaaa tgcataagct
tttgccattc
```

541 tcaccggatt cagtcgtcac tcatggtgat ttctcacttg ataaccttat ttttgacgag
601 gggaaattaa taggttgtat tgatgttgga cgagtcggaa tcgcagaccg ataccaggat
661 cttgccatcc tatggaactg cctcggtgag ttttctcctt cattacagaa acggcttttt
721 caaaaatatg gtattgataa tcctgatatg aataaattgc agtttcattt gatgctcgat
781 gagtttttct aaatcagaat tggttaattg gttgtaacac tggcactcaa ccaagtcatt
841 ctgagaatag tgtatgcggc gaccgagttg ctcttgcccg gcgtcaatac gggatGCTTT
901 CCACTTATTC AACCTTTTAT AGCCTAAACG TCGTATAGGA GCATTTCCGC GGAGACAGAT
961 CGCTGAGATA GGTGCCTCAC TGATTAAGCA TTGGTAACTG TCAGACCAAG TTTACTCATA
1021 TATACTTTAG ATTGATTTAA AACTTCATTT TTAATTTAAA AGGATCTAGG TGAAGATCCT
1081 TTTTGATAAT CTCATGACCA AAATCCCTTA ACGTGAGTTT TCGTTCCACT GAGCGTCAGA
1141 CCCCGTAGAA AAGATCAAAG GATCTTCTTG AGATCCTTTT TTTCTGCGCG TAATCTGCTG
1201 CTTGCAAACA AAAAAACCAC CGCTACCAGC GGTGGTTTGT TTGCCGGATC AAGAGCTACC
1261 AACTCTTTTT CCGAAGGTAA CTGGCTTCAG CAGAGCGCAG ATACCAAATA CTGTCCTTCT
1321 AGTGTAGCCG TAGTTAGGCC ACCACTTCAA GAACTCTGTA GCACCGCCTA CATACCTCGC
1381 TCTGCTAATC CTGTTACCAG TGGCTGCTGC CAGTGGCGAT AAGTCGTGTC TTACCGGGTT
1441 GGACTCAAGA CGATAGTTAC CGGATAAGGC GCAGCGGTCG GGCTGAACGG GGGGTTCGTG
1501 CACACAGCCC AGCTTGGAGC GAACGACCTA CACCGAACTG AGATACCTAC AGCGTGAGCA
1561 TTGAGAAAGC GCCACGCTTC CCGAAGGGAG AAAGGCGGAC AGGTATCCGG TAAGCGGCAG
1621 GGTCGGAACA GGAGAGCGCA CGAGGGAGCT TCCAGGGGGA AACGCCTGGT ATCTTTATAG
1681 TCCTGTCGGG TTTCGCCACC TCTGACTTGA GCGTCGATTT TTGTGATGCT CGTCAGGGGG
1741 GCGGAGCCTA TGGAAAAACG CCAGCAACGC GGCCTTTTTA CGGTTCCTGG CCTTTTGCTG
1801 GCCTTTTGCT CACATGTTCT TTCCTGCGTT ATCCCCTGAT TCTGTGGATA ACCGTATTAC
1861 CGCCTTTGAG TGAGCTGATA CCGCTCGCCG CAGCCGAACG ACCGAGCGCA GCGAGTCAGT
1921 GAGCGAGGAA GCGGAAGAGC GCCCAATACG CAAACCGCCT CTCCCCGCGC GTTGGCCGAT
1981 TCATTAATGC AGGTTGACGT CGCGCAACGC AATTAATGTG AGTTAGCTCA CTCATTAGGC
2041 ACCCTTGAGG AGGTTTCTCT GTTAATccag gcatcaaata aaacgaaagg ctcagtcgaa
2101 agactgggcc tttcgtttta tctgttgttt gtcggtgaac gctctctact agagtcacac

2161 tggctcacct tcgggtgggc ctttctgcgt ttatagCATC ACGTGCTATA AAAATAATTA
2221 TAATTTAAAT TTTTTAATAT AAATATATAA ATTAAAAATA GAAAGTAAAA AAAGAAATTA
2281 AAGAAAAAAT AGTTTTTGTT TTCCGAAGAT GTAAAAGACT CTAGGGGGAT CGCCAACAAA
2341 TACTACCTTT TATCTTGCTC TTCCTGCTCT CAGGTATTAA TGCCGAATTG TTTCATCTTG
2401 TCTGTGTAGA AGACCACACA CGAAAATCCT GTGATTTTAC ATTTTACTTA TCGTTAATCG
2461 AATGTATATC TATTTAATCT GCTTTTCTTG TCTAATAAAT ATATATGTAA AGTACGCTTT
2521 TTGTTGAAAT TTTTTAAACC TTTGTTTATT TTTTTTTCTT CATTCCGTAA CTCTTCTACC
2581 TTCTTTATTT ACTTTCTAAA ATCCAAATAC AAAACATAAA AATAAATAAA CACAGAGTAA
2641 ATTCCCAAAT TATTCCATCA TTAAAAGATA CGAGGCGCGT GTAAGTTACA GGCAAGCGAT
2701 CCTAGTACAC TCTATATTTT TTTATGCCTC GGTAATGATT TTCATTTTTT TTTTTCCACC
2761 TAGCGGATGA CTCTTTTTTT TTCTTAGCGA TTGGCATTAT CACATAATGA ATTATACATT
2821 ATATAAAGTA ATGTGATTTC TTCGAAGAAT ATACTAAAAA ATGAGCAGGC AAGATAAACG
2881 AAGGCAAAGA TGACAGAGCA GAAAGCCCTA GTAAAGCGTA TTACAAATGA AACCAAGATT
2941 CAGATTGCGA TCTCTTTAAA GGGTGGTCCC CTAGCGATAG AGCACTCGAT CTTCCCAGAA
3001 AAAGAGGCAG AAGCAGTAGC AGAACAGGCC ACACAATCGC AAGTGATTAA CGTCCACACA
3061 GGTATAGGGT TTCTGGACCA TATGATACAT GCTCTGGCCA AGCATTCCGG CTGGTCGCTA
3121 ATCGTTGAGT GCATTGGTGA CTTACACATA GACGACCATC ACACCACTGA AGACTGCGGG
3181 ATTGCTCTCG GTCAAGCTTT TAAAGAGGCC CTACTGGCGC GTGGAGTAAA AAGGTTTGGA
3241 TCAGGATTTG CGCCTTTGGA TGAGGCACTT TCCAGAGCGG TGGTAGATCT TTCGAACAGG
3301 CCGTACGCAG TTGTCGAACT TGGTTTGCAA AGGGAGAAAG TAGGAGATCT CTCTTGCGAG
3361 ATGATCCCGC ATTTTCTTGA AAGCTTTGCA GAGGCTAGCA GAATTACCCT CCACGTTGAT
3421 TGTCTGCGAG GCAAGAATGA TCATCACCGT AGTGAGAGTG CGTTCAAGGC TCTTGCGGTT
3481 GCCATAAGAG AAGCCACCTC GCCCAATGGT ACCAACGATG TTCCCTCCAC CAAAGGTGTT
3541 CTTATGTAGT TTTACACAGG AGTCTGGACT TGAtcgattt attcaacaaa gccacgttgt
3601 gtctcaaaat ctctgatgtt acattgcaca agataaaaat atatcatcat gaacaataaa
3661 actgtctgct tacataaaca gtaatacaag ggtgttatg agccatattc aacgggaaac
3721 g
//
