# Supplementary material for: Genetic Entanglement Enables Ultrastable Biocontainment in the Mammalian Gut
Source: ACS Synth Biol. 2025 Sep 7;14(9):3696–708. doi: 10.1021/acssynbio.5c00412 (PMC12455656; doi:10.1021/acssynbio.5c00412)
Supplement: Supplementary file 9 [file sb5c00412_si_009.pdf]

```
LOCUS       I-OnuI-533(Im9)_w_colE9 7550 bp ds-DNA     circular     08-
MAY-2025
DEFINITION  .
KEYWORDS    "creator:SnapGene License" "marker:AmpR"
FEATURES             Location/Qualifiers
     promoter        909..943
                     /label="J23108"
                     /ApEinfo_revcolor="#c6c9d1"
                     /ApEinfo_fwdcolor="#c6c9d1"
                     /note="note: bacterial promoter (Registry of
Standard Biological Parts BBa_J23119)"
     RBS             975..997
                     /label="RBS"
                     /ApEinfo_revcolor="#b4abac"
                     /ApEinfo_fwdcolor="#b4abac"
                     /note="note: efficient ribosome binding site from
bacteriophage T7 gene 10 (Olins and Rangwala, 1989)"
     CDS             1005..3284
                     /label="Onu TSM (site 2), ribsored"
                     /ApEinfo_revcolor="#75c6a9"
                     /ApEinfo_fwdcolor="#75c6a9"

/translation="MGSAYMSRRESINPWILTGFADAEGSFLLRIRNNNKSSVGYSTELGFQITLHNKDKSIL
ENIQSTWKVGVIANSGDNAVSLKVTRFEDLKVIIDHFEKYPLITQKLGDYMLFKQAFCVMENKEHLKINGIKE
LVRIKAKLNWGLTDELKKAFPEIISKERSLINKNIPNFKWLAGFTSGEGCFFVNLIKSKSKLGVQVQLVFSIT
QHIKDKNLMNSLITYLGCFAKGTNVLMADGSIECIENIEVGNKVMGKDGRPREVIKLPRGRETMYSVVQKSQH
RAHKSDSSREVPELLKFTCNATHELVVRTPRSVRRLSRTIKGVEYFEVITFEMGQKKAPDGRIVELVKEVSKS
YPISEGPERANELVESYRKASNKAYFEWTIEARDLSLLGSHVRKATYQTYAPILYENDHFFDYMQKSKFHLTI
EGPKVLAYLPGLWIGDGLSDRATFSVDSRDTSLMERVTEYAEKLNLCAEYKDRKEPQVAKTVNLYSKVVRGNG
IRNNLNTENPLWDAIVGLGFLKDGVKNIPSFLSTDNIGTRETFLAGLIDSDGYVTDEHGIKATIKTIHTSVRD
GLVSLARSLGLVVSVNAEPAKVDMNGTKHKISYAIYMSGGDVLLNVLSKCAGSKKFRPAPAAAFARECRGFYF
ELQELKEDDYYGITLSDDSDHQFLLANQVVVHNCGYIKEKNKSEFSWLDFVVTKFSDINDKIIPVFQENTLIG
VKLEDFEDWCKVAKLIEEKKHLTESGLDEIKKIKLNMNKGRVF*"
     "altered" RBS   1538..1543
                     /label="RBS"
                     /ApEinfo_revcolor="#b4abac"
                     /ApEinfo_fwdcolor="#b4abac"
     CDS             1547..3598
                     /label="entangled reading frame"
                     /ApEinfo_revcolor="#84b0dc"
                     /ApEinfo_fwdcolor="#84b0dc"

/translation="MLLRESYQEQEQTGRASSTGIQYHTAYQGQESDEQPYHISGLFREGHKRADGGRQHRVY
REYRSGQQSDGQGRASAGSDQAAAWAGDHVQRGSEEPTPGTQVRQQPGGSGAAEIHLQRHTRTGGAYTAQRAA
PEPHNQGRGVLRGDHLRNGSEEGTGWPYRGTGEGGEQVISDLRGSGTGKRAGGELPQSQQQGLLRMDHRSTGP
EPSGQPRAQGHISNLRTYPVRERSFLRLYAEEQIPPDNRGTEGAGLPAGPLDRRRSVRPGHLQRGQPGHQPDG
ARDGVRGETEPLCGIQGPQGAAGGKDREPVQQGGAWQRHPQQPEHGESALGRHRGSGLPEGWREEHTELSEHG
QHWHPGDLPGRPDRLRRLRDGRTRHQGHHQDDPHQRAGRPGEPGPQPGSGGERERGAGQSGYERYKAQDQLRH
LHERRRRAAERAEQMCWQQEVPSGTGSRLRPGMSWFLLRTAGAEGGRLLRYHPVRRLRPPIPAGKSSGGAQLW
LYQGEEQVRIQLAGLRGDQVLRYQRQDHTSVPGEYPDWRQAGGFRGLVQSGQTYRGEETSYGERSGRNQEDQT
QYEQGPGVLSYPYDVPDYAGGGSGGGSMELKHSISDYTEAEFLQLVTTICNADTSSEEELVKLVTHFEEMTEH
PSGSDLIYYPKEGDDDSPSGIVNTVKQWRAANGKSGFKQG*"
     CDS             3285..3337
                     /label="GS linker"
```

```
                     /ApEinfo_revcolor="#993366"
                     /ApEinfo_fwdcolor="#993366"
                     /note="codon_start: 3 transl_table: 1"
     misc_feature    3287..3313
                     /label="HA-tag"
                     /ApEinfo_revcolor="#f8d3a9"
                     /ApEinfo_fwdcolor="#f8d3a9"
     CDS             3338..3598
                     /label="Im9"
                     /ApEinfo_revcolor="#ff9ccd"
                     /ApEinfo_fwdcolor="#ff9ccd"

/translation="MELKHSISDYTEAEFLQLVTTICNADTSSEEELVKLVTHFEEMTEHPSGSDLIYYPKEG
DDDSPSGIVNTVKQWRAANGKSGFKQG*"
     terminator      3621..3894
                     /label="Aph 3' Terminator"
                     /ApEinfo_revcolor="#b4abac"
                     /ApEinfo_fwdcolor="#b4abac"
     terminator      complement(4230..4257)
                     /label="T7Te terminator"
                     /ApEinfo_revcolor="#c6c9d1"
                     /ApEinfo_fwdcolor="#c6c9d1"
                     /note="note: phage T7 early transcription
terminator"
     terminator      complement(4273..4344)
                     /label="rrnB T1 terminator"
                     /ApEinfo_revcolor="#c6c9d1"
                     /ApEinfo_fwdcolor="#c6c9d1"
                     /note="note: transcription terminator T1 from the E.
coli rrnB gene"
     CDS             complement(4389..6155)
                     /label="colE9 K12 optimized"
                     /ApEinfo_revcolor="#faac61"
                     /ApEinfo_fwdcolor="#faac61"

/translation="MHHHHHHSGGDGRGHNTGAHSTSGNINGGPTGIGVSGGASDGSGWSSENNPWGGGSGSG
IHWGGGSGRGNGGGNGNSGGGSGTGGNLSAVAAPVAFGFPALSTPGAGGLAVSISASELSAAIAGIIAKLKKV
NLKFTPFGVVLSSLIPSEIAKDDPNMMSKIVTSLPADDITESPVSSLPLDKATVNVNVRVVDDVKDERQNISV
VSGVPMSVPVVDAKPTERPGVFTASIPGAPVLNISVNDSTPAVQTLSPGVTNNTDKDVRPAGFTQGGNTRDAV
IRFPKDSGHNAVYVSVSDVLSPDQVKQRQDEENRRQQEWDATHPVEAAERNYERARAELNQANEDVARNQERQ
AKAVQVYNSRKSELDAANKTLADAIAEIKQFNRFAHDPMAGGHRMWQMAGLKAQRAQTDVNNKQAAFDAAAKE
KSDADAALSAAQERRKQKENKEKDAKDKLDKESKRNKPGKATGKGKPVGDKWLDDAGKDSGAPIPDRIADKLR
DKEFKSFDDFRKAVWEEVSKDPELSKNLNPSNKSSVSKGYSPFTPKNQQVGGRKVYELHHDKPISQGGEVYDM
DNIRVTTPKRHIDIHRGK*"
     misc_feature    6163..6176
                     /label="B0031"
                     /ApEinfo_revcolor="#b4abac"
                     /ApEinfo_fwdcolor="#b4abac"
     misc_feature    complement(6198..6232)
                     /label="J23106"
                     /ApEinfo_revcolor="#c7b0e3"
                     /ApEinfo_fwdcolor="#c7b0e3"
     promoter        6465..6569
                     /label="AmpR promoter"
```

```
                     /ApEinfo_revcolor="#c6c9d1"
                     /ApEinfo_fwdcolor="#c6c9d1"
                     /note="gene: bla"
     CDS             6570..7221
                     /label="AmpR"
                     /ApEinfo_revcolor="#993366"
                     /ApEinfo_fwdcolor="#993366"
                     /note="codon_start: 1 transl_table: 1"
     rep_origin      7386..381
                     /label="p15A ori"
                     /ApEinfo_revcolor="#ffef86"
                     /ApEinfo_fwdcolor="#ffef86"
                     /note="direction: RIGHT note: Plasmids containing
the medium-copy-number p15A origin of replication can be propagated in E.
coli cells that contain a second plasmid with the ColE1 origin."
ORIGIN
        1 GGCGCATGAC TTCAAGACTA ACTCCTCTAA ATCAATTACC AGTGGCTGCT
GCCAGTGGTG
       61 CTTTTGCATG TCTTTCCGGG TTGGACTCAA GACGATAGTT ACCGGATAAG
GCGCAGCGGT
      121 CGGACTGAAC GGGGGGTTCG TGCATACAGT CCAGCTTGGA GCGAACTGCC
TACCCGGAAC
      181 TGAGTGTCAG GCGTGGAATG AGACAAACGC GGCCATAACA GCGGAATGAC
ACCGGTAAAC
      241 CGAAAGGCAG GAACAGGAGA GCGCACGAGG GAGCCGCCAG GGGGAAACGC
CTGGTATCTT
      301 TATAGTCCTG TCGGGTTTCG CCACCACTGA TTTGAGCGTC AGATTTCGTG
ATGCTTGTCA
      361 GGGGGGCGGA GCCTATGGAA AAACGGCTTT GCCGCGGCCC TCTCACTTCC
CTGTTAAGTA
      421 TCTTCCTGGC ATCTTCCAGG AAATCTCCGC CCCGTTCGTA AGCCATTTCC
GCTCGCCGCA
      481 GTCGAACGAC CGAGCGTAGC GAGTCAGTGA GCGAGGAAGC GGAATATATC
CTGTATCACA
      541 TATTCTGCTG ACGCACCGGT GCAGCCTTTT TTCTCCTGCC ACATGAAGCA
CTTCACTGAC
      601 ACCCTCATCA GTGCCAACAT AGTAAGCCAG TATACACTCC GCTAGCGCTG
AGGTCTGCCT
      661 CGTGAAGAAG GTGTTGCTGA CTCATACCAG GCCTGAATCG CCCCATCATC
CAGCCAGAAA
      721 GTGAGGGAGC CACGGTTGAT GAGAGCTTTG TTGTAGGTGG ACCAGTTGGT
GATTTTGAAC
      781 TTTTGCTTTG CCACGGAACG GTCTGCGTTG TCGGCATGCG CATAATGTGC
CTGTCAAATG
      841 GACGAAGCAG GGATTCTGCA AACCCTATGC TACTCCGTCA AGCCGTCAAT
TGTCTGATTC
      901 GTTACCAAct gacagctagc tcagtcctag gtataatgct agcTCCATAC
CCGTTTTTTT
      961 GGGCTAGAAA TAATTTTGTT TAACTTTAAG AAGGAGATAT ACCCATGGGT
AGCGCATATA
     1021 TGAGCCGCCG TGAAAGCATC AATCCCTGGA TCCTGACAGG CTTTGCAGAT
GCCGAGGGTA
     1081 GCTTCCTGCT TCGTATTCGT AATAATAATA AGAGCAGTGT GGGTTATAGC
ACCGAACTGG
```

1141 GCTTCCAGAT CACCCTGCAC AATAAAGATA AGTCTATCCT GGAGAACATT CAAAGCACCT
1201 GGAAAGTTGG TGTTATTGCA AATAGTGGCG ATAATGCAGT TAGCCTTAAA GTGACCCGCT
1261 TCGAAGATCT TAAAGTGATC ATTGATCACT TCGAGAAGTA TCCGCTTATC ACCCAGAAGC
1321 TGGGCGATTA TATGCTCTTC AAACAAGCCT TCTGTGTGAT GGAGAACAAA GAACACCTGA
1381 AGATCAACGG CATTAAAGAA CTTGTTCGTA TCAAAGCCAA ACTGAACTGG GGTCTTACCG
1441 ACGAACTTAA GAAGGCATTC CCAGAAATTA TTAGCAAAGA ACGTAGCCTG ATTAATAAGA
1501 ACATTCCGAA CTTTAAATGG CTTGCCGGCT TCACCTCAGG AGAGGGATGC TTCTTCGTGA
1561 ATCTTATCAA GAGCAAGAGC AAACTGGGCG TGCAAGTTCA ACTGGTATTC AGTATCACAC
1621 AGCATATCAA GGACAAGAAT CTGATGAACA GCCTTATCAC ATATCTGGGC TGTTTCGCGA
1681 AGGGCACAAA CGTGCTGATG GCGGACGGCA GCATAGAGTG TATAGAGAAT ATAGAAGTGG
1741 GCAACAAAGT GATGGGCAAG GACGGGCGTC CGCGGGAAGT GATCAAGCTG CCGCGTGGGC
1801 GGGAGACCAT GTACAGCGTG GTTCAGAAGA GCCAACACCG GGCACACAAG TCAGACAGCA
1861 GCCGGGAGGT TCCGGAGCTG CTGAAATTCA CCTGCAACGC CACACACGAA CTGGTGGTGC
1921 GTACACCGCG CAGCGTGCGG CGCCTGAGCC GCACAATCAA GGGCGTGGAG TACTTCGAGG
1981 TGATCACCTT CGAAATGGGT CAGAAGAAGG CACCGGATGG CCGTATCGTG GAACTGGTGA
2041 AGGAGGTGAG CAAGTCATAT CCGATCTCAG AGGGTCCGGA ACGGGCAAAC GAGCTGGTGG
2101 AGAGTTACCG CAAAGCCAGC AACAAGGCTT ACTTCGAATG GACCATAGAA GCACGGGACC
2161 TGAGCCTTCT GGGCAGCCAC GTGCGCAAGG CCACATATCA AACTTACGCA CCTATCCTGT
2221 ACGAGAACGA TCATTTCTTC GATTATATGC AGAAGAGCAA ATTCCACCTG ACAATAGAGG
2281 GACCGAAGGT GCTGGCTTAC CTGCCGGGCC TCTGGATCGG AGACGGTCTG TCAGACCGGG
2341 CCACCTTCAG CGTGGACAGC CGGGACACCA GCCTGATGGA GCGCGTGACG GAGTACGCGG
2401 AGAAACTGAA CCTCTGTGCG GAATACAAGG ACCGCAAGGA GCCGCAGGTG GCAAAGACCG
2461 TGAACCTGTA CAGCAAGGTG GTGCGTGGCA ACGGCATCCG CAACAACCTG AACACGGAGA
2521 ATCCGCTCTG GGACGCCATC GTGGGTCTGG GCTTCCTGAA GGATGGCGTG AAGAACATAC
2581 CGAGCTTTCT GAGCACGGAC AACATTGGCA CCCGGGAGAC CTTCCTGGCC GGCCTGATAG
2641 ACTCAGACGG TTACGTGACG GACGAACACG GCATCAAGGC CACCATCAAG ACGATCCACA
2701 CCAGCGTGCG GGACGGCCTG GTGAGCCTGG CCCGCAGCCT GGGTCTGGTG GTGAGCGTGA

2761 ACGCGGAGCC GGCCAAAGTG GATATGAACG GTACAAAGCA CAAGATCAGT TACGCCATTT
2821 ACATGAGCGG CGGAGACGTG CTGCTGAACG TGCTGAGCAA ATGTGCTGGC AGCAAGAAGT
2881 TCCGTCCGGC ACCGGCAGCC GCCTTCGCCC GGGAATGTCG TGGTTTCTAC TTCGAACTGC
2941 AGGAGCTGAA GGAGGACGAT TATTACGGTA TCACCCTGTC AGACGACTCA GACCACCAAT
3001 TCCTGCTGGC AAATCAAGTG GTGGTGCACA ACTGTGGTTA TATCAAGGAG AAGAACAAGT
3061 CAGAATTCAG CTGGCTGGAC TTCGTGGTGA CCAAGTTCTC AGATATCAAC GACAAGATCA
3121 TACCAGTGTT CCAGGAGAAT ACCCTGATTG GCGTCAAGCT GGAGGATTTC GAGGACTGGT
3181 GCAAAGTGGC CAAACTTATA GAGGAGAAGA AACATCTTAC GGAGAGCGGT CTGGACGAAA
3241 TCAAGAAGAT CAAACTCAAT ATGAACAAGG GCCGGGTGTT CTGAGCTATC CATACGATGT
3301 CCCGGACTAC GCTGGTGGCG GTAGCGGTGG AGGTAGTATG GAACTGAAAC ATAGCATTAG
3361 CGATTATACC GAAGCCGAAT TTCTGCAGCT GGTGACCACC ATTTGTAATG CCGATACCAG
3421 CAGCGAAGAA GAACTGGTGA AACTGGTGAC CCATTTTGAA GAAATGACCG AACATCCGAG
3481 CGGTAGCGAT CTGATTTATT ATCCGAAAGA AGGTGATGAT GATAGCCCGA GCGGTATTGT
3541 GAATACCGTG AAACAGTGGC GTGCCGCCAA TGGTAAAAGC GGTTTTAAAC AGGGTTAAGC
3601 GGCCGCTCAG AATTGGTTAA TTGGTTGTAA CACTGGCAGA GCATTACGCT GACTTGACGG
3661 GACGGCGGCT TTGTTGAATA AATCGAACTT TTGCTGAGTT GAAGGATCAG ATCACGCATC
3721 TTCCCGACAA CGCAGACCGT TCCGTGGCAA AGCAAAAGTT CAAAATCACC AACTGGTCCA
3781 CCTACAACAA AGCTCTCATC AACCGTGGCT CCCTCACTTT CTGGCTGGAT GATGGGGCGA
3841 TTCAGGCCTG GTATGAGTCA GCAACACCTT CTTCACGAGG CAGACCTCAG CGCTCAAAGA
3901 TGCAGGGGTA AAAGCTAACC GCATCTTTAC CGACAAGGCA TCCGGCAGTT CAACAGATCG
3961 GGAAGGGCTG GATTTGCTGA GGATGAAGGT GGAGGAAGGT GATGTCATTC TGGTGAAGAA
4021 GCTCGACCGT CTTGGCCGCG ACACCGCCGA CATGATCCAA CTGATAAAAG AGTTTGATGC
4081 TCAGGGTGTA GCGGTTCGGT TTATTGACGA CGGGATCAGT ACCGACGGTG ATATGGGGCA
4141 AATGGTGGTC ACCATCCTGT CGGCTGTGGC ACAGGCTGAA CGCCGGAGGA TCCTAGAGCG
4201 CACGAATGAG GGCCGACAGG AAGCAAAGCc gcagaaaggc ccacccgaag gtgagccagt
4261 gtgactctag tagagagcgt tcaccgacaa acaacagata aaacgaaagg cccagtcttt
4321 cgactgagcc tttcgtttta tttgatgcct ggctctagta gcgatctaca ctagcactat

4381 cagcgttaTT ATTTACCACG GTGGATGTCG ATGTGGCGTT TGGGAGTCGT GACACGGATG
4441 TTGTCCATAT CGTACACCTC CCCTCCCTGC GAAATTGGTT TGTCGTGGTG CAGTTCGTAG
4501 ACTTTGCGGC CGCCCACCTG TTGGTTTTTC GGAGTAAACG GAGAATAACC CTTTGAGACC
4561 GAACTCTTGT TGCTGGGATT TAAGTTTTTG CTTAATTCCG GATCTTTGCT TACTTCCTCC
4621 CATACGGCTT TGCGAAAATC ATCGAATGAC TTGAACTCCT TGTCACGTAA TTTATCTGCA
4681 ATACGGTCGG GGATTGGAGC GCCACTATCC TTGCCTGCGT CATCCAGCCA TTTGTCACCA
4741 ACGGGTTTGC CTTTCCCGGT CGCTTTTCCA GGCTTGTTAC GCTTAGACTC CTTGTCCAGC
4801 TTGTCTTTAG CGTCTTTCTC CTTATTTTCT TTTTGTTTGC GGCGTTCTTG TGCTGCTGAC
4861 AATGCAGCGT CCGCATCTGA TTTCTCCTTG GCTGCAGCAT CGAAAGCAGC TTGCTTATTG
4921 TTGACGTCGG TCTGGGCGCG TTGCGCCTTT AAGCCTGCCA TCTGCCACAT GCGGTGGCCG
4981 CCAGCCATTG GATCGTGCGC GAAGCGATTG AACTGCTTAA TCTCAGCGAT GGCGTCTGCC
5041 AAAGTTTTAT TGGCGGCATC AAGCTCAGAT TTACGGGAAT TATACACTTG GACGGCTTTC
5101 GCTTGGCGTT CTTGATTACG AGCCACGTCT TCGTTAGCCT GATTTAACTC AGCGCGTGCA
5161 CGTTCATAGT TACGTTCAGC GGCTTCTACG GGGTGCGTAG CATCCCATTC CTGTTGACGG
5221 CGGTTTTCTT CATCCTGACG TTGTTTAACT TGATCTGGAG ACAGCACATC TGAAACCGAA
5281 ACGTACACCG CGTTATGGCC AGAATCCTTG GGGAAGCGGA TAACCGCATC GCGTGTGTTA
5341 CCACCCTGCG TAAACCCAGC TGGACGAACA TCCTTATCTG TATTGTTCGT AACTCCGGGC
5401 GATAAGGTCT GGACAGCCGG AGTCGAGTCA TTCACTGAAA TGTTCAGCAC TGGTGCTCCG
5461 GGGATGGAGG CTGTGAATAC ACCTGGACGC TCTGTTGGTT TGGCGTCAAC CACGGGGACA
5521 CTCATCGGGA CCCCGCTAAC TACCGAAATA TTCTGGCGTT CATCCTTTAC GTCATCGACG
5581 ACACGAACGT TAACGTTCAC TGTCGCCTTA TCAAGCGGCA GAGAGGAAAC GGGGGATTCG
5641 GTAATATCAT CCGCCGGCAG ACTGGTGACA ATTTTGCTCA TCATGTTGGG ATCATCCTTG
5701 GCGATTTCTG ACGGAATCAA ACTAGACAGA ACAACGCCGA AGGGCGTAAA TTTAAGATTT
5761 ACCTTCTTTA ACTTCGCGAT GATTCCGGCA ATTGCGGCGG ACAGCTCGGA CGCCGAAATG
5821 GACACCGCCA AGCCACCTGC GCCGGGCGTA GAAAGCGCTG GGAAACCGAA CGCGACAGGC
5881 GCTGCTACTG CGGATAAATT TCCTCCAGTC CCGGAGCCGC CTCCACTATT ACCGTTTCCT
5941 CCCCCGTTTC CGCGCCCGCT CCCTCCTCCC CAGTGGATAC CCGATCCGGA GCCCCCACCC

```
     6001 CAAGGGTTGT TCTCGGAAGA CCATCCCGAG CCATCAGATG CCCCACCGCT
GACCCCGATC
     6061 CCCGTCGGTC CCCCATTGAT ATTACCGCTT GTGCTGTGGG CGCCAGTGTT
GTGACCACGT
     6121 CCGTCACCCC CTGAATGGTG GTGGTGATGA TGCATGGGTA TAggtttcct
gtgtgactct
     6181 agtatgGAAT TCtgtgagct agcactatac ctaggactga gctagccgta
aaTGAAAGGA
     6241 ATCAAATTTG GCCGCAGGCG TACCGTGGAC AGGAACGTCG TGCTGACGCT
TCATCAGAAG
     6301 GGCACTGGTG CAACGGAAAT TGCTCATCAG CTCAGTATTG CCCGCTCCAC
GGTTTATAAA
     6361 ATTCTTGAAG ACGAAAGGGC CTCGTGATAC GCCTATTTTT ATAGGTTAAT
GTCATGATAA
     6421 TAATGGTTTC TTAGACGTCA GGTGGCACTT TTCGGGGAAA TGTGCGCGGA
ACCCCTATTT
     6481 GTTTATTTTT CTAAATACAT TCAAATATGT ATCCGCTCAT GAGACAATAA
CCCTGATAAA
     6541 TGCTTCAATA ATATTGAAAA AGGAAGAGTA TGAGTATTCA ACATTTCCGT
GTCGCCCTTA
     6601 TTCCCTTTTT TGCGGCATTT TGCCTTCCTG TTTTTGCTCA CCCAGAAACG
CTGGTGAAAG
     6661 TAAAAGATGC TGAAGATCAG TTGGGTGCAC GAGTGGGTTA CATCGAACTG
GATCTCAACA
     6721 GCGGTAAGAT CCTTGAGAGT TTTCGCCCCG AAGAACGTTT TCCAATGATG
AGCACTTTTA
     6781 AAGGGACCGA AGGAGCTAAC CGCTTTTTTG CACAACATGG GGGATCATGT
AACTCGCCTT
     6841 GATCGTTGGG AACCGGAGCT GAATGAAGCC ATACCAAACG ACGAGCGTGA
CACCACGATG
     6901 CCTGCAGCAA TGGCAACAAC GTTGCGCAAA CTATTAACTG GCGAACTACT
TACTCTAGCT
     6961 TCCCGGCAAC AATTAATAGA CTGGATGGAG GCGGATAAAG TTGCAGGACC
ACTTCTGCGC
     7021 TCGGCCCTTC CGGCTGGCTG GTTTATTGCT GATAAATCTG GAGCCGGTGA
GCGTGGGTCT
     7081 CGCGGTATCA TTGCAGCACT GGGGCCAGAT GGTAAGCCCT CCCGTATCGT
AGTTATCTAC
     7141 ACGACGGGGA GTCAGGCAAC TATGGATGAA CGAAATAGAC AGATCGCTGA
GATAGGTGCC
     7201 TCACTGATTA AGCATTGGTA ACTGTCAGAC CAAGTTTACT CATATATACT
TTAGATTGAT
     7261 TTAAAACTTC ATTTTTAATT TAAAAGGATC TAGGTGAAGA TCCTTTTTGA
TAATCTCATG
     7321 ACCAAAATCC CTTAACGTGA GTTTTCGTTC CACTGAGCGT CAGACCCCTT
AATAAGATGA
     7381 TCTTCTTGAG ATCGTTTTGG TCTGCGCGTA ATCTCTTGCT CTGAAAACGA
AAAAACCGCC
     7441 TTGCAGGGCG GTTTTTCGAA GGTTCTCTGA GCTACCAACT CTTTGAACCG
AGGTAACTGG
     7501 CTTGGAGGAG CGCAGTCACC AAAACTTGTC CTTTCAGTTT AGCCTTAACC
//
```
