# Supplementary material for: Genetic Entanglement Enables Ultrastable Biocontainment in the Mammalian Gut
Source: ACS Synth Biol. 2025 Sep 7;14(9):3696–708. doi: 10.1021/acssynbio.5c00412 (PMC12455656; doi:10.1021/acssynbio.5c00412)
Supplement: Supplementary file 10 [file sb5c00412_si_010.pdf]

```
LOCUS       pEndo_I-PanMI-126(Im9/c 7523 bp ds-DNA     circular     04-
MAR-2025
DEFINITION  .
KEYWORDS    "creator:SnapGene License" "marker:AmpR"
FEATURES             Location/Qualifiers
     promoter        909..943
                     /label="J23108 promoter"
                     /ApEinfo_revcolor="#c6c9d1"
                     /ApEinfo_fwdcolor="#c6c9d1"
                     /note="note: bacterial promoter (Registry of
Standard Biological Parts BBa_J23119)"
     RBS             975..997
                     /label="RBS"
                     /ApEinfo_revcolor="#b4abac"
                     /ApEinfo_fwdcolor="#b4abac"
                     /note="note: efficient ribosome binding site from
bacteriophage T7 gene 10 (Olins and Rangwala, 1989)"
     CDS             1005..3284
                     /label="Pan TSM (site 1), ribosored"
                     /ApEinfo_revcolor="#339966"
                     /ApEinfo_fwdcolor="#339966"
                     /note="codon_start: 1 transl_table: 1 translation:
MGFKRNFSTLESKLNPSYISGFVDGEGSFMLTIIKDNKYKLGRRVVCRFVISLHKKDLSLLNKIKEFFDVGNV
FLMTKDSAQYRVESLKGLDLIINHFDKYPLITKKQADYKLFKMAHNLIKNKSHLTKEGLLELVAIKAVINNGL
NNDLSIAFPGINTILRPDTSLPQILNPFWLSGFVDAEGCFAKGTNVLMADGSIECIENIEVGNKVMGKDGRPR
EVIKLPRGRETMYSVVQKSQHRAHKSDSSREVPELLKFTCNATHELVVRTPRSVRRLSRTIKGVEYFEVITFE
MGQKKAPDGRIVELVKEVSKSYPISEGPERANELVESYRKASNKAYFEWTIEARDLSLLGSHVRKATYQTYAP
ILYENDHFFDYMQKSKFHLTIEGPKVLAYLPGLWIGDGLSDRATFSVDSRDTSLMERVTEYAEKLNLCAEYKD
RKEPQVAKTVNLYSKVVRGNGIRNNLNTENPLWDAIVGLGFLKDGVKNIPSFLSTDNIGTRETFLAGLIDSDG
YVTDEHGIKATIKTIHTSVRDGLVSLARSLGLVVSVNAEPAKVDMNGTKHKISYAIYMSGGDVLLNVLSKCAG
SKKFRPAPAAAFARECRGFYFELQELKEDDYYGITLSDDSDHQFLLANQVVVHNCFSVVVFKSKTSKLGEAVK
LSFILTQSNRDEYLIKSLIEYLGCGNTSLDPRGTIDFKVTNFSSIKDIIVPFFIKYPLKGNKNLDFTDFCEVV
RLMENKSHLTKEGLDQIKKIRNRMNTNRK*"
     CDS             1005..3284
                     /ApEinfo_revcolor="#84b0dc"
                     /ApEinfo_fwdcolor="#84b0dc"

/translation="MGFKRNFSTLESKLNPSYISGFVDGEGSFMLTIIKDNKYKLGRRVVCRFVISLHKKDLS
LLNKIKEFFDVGNVFLMTKDSAQYRVESLKGLDLIINHFDKYPLITKKQADYKLFKMAHNLIKNKSHLTKEGL
LELVAIKAVINNGLNNDLSIAFPGINTILRPDTSLPQILNPFWLSGFVDAEGCFAKGTNVLMADGSIECIENI
EVGNKVMGKDGRPREVIKLPRGRETMYSVVQKSQHRAHKSDSSREVPELLKFTCNATHELVVRTPRSVRRLSR
TIKGVEYFEVITFEMGQKKAPDGRIVELVKEVSKSYPISEGPERANELVESYRKASNKAYFEWTIEARDLSLL
GSHVRKATYQTYAPILYENDHFFDYMQKSKFHLTIEGPKVLAYLPGLWIGDGLSDRATFSVDSRDTSLMERVT
EYAEKLNLCAEYKDRKEPQVAKTVNLYSKVVRGNGIRNNLNTENPLWDAIVGLGFLKDGVKNIPSFLSTDNIG
TRETFLAGLIDSDGYVTDEHGIKATIKTIHTSVRDGLVSLARSLGLVVSVNAEPAKVDMNGTKHKISYAIYMS
GGDVLLNVLSKCAGSKKFRPAPAAAFARECRGFYFELQELKEDDYYGITLSDDSDHQFLLANQVVVHNCFSVV
VFKSKTSKLGEAVKLSFILTQSNRDEYLIKSLIEYLGCGNTSLDPRGTIDFKVTNFSSIKDIIVPFFIKYPLK
GNKNLDFTDFCEVVRLMENKSHLTKEGLDQIKKIRNRMNTNRK*"
     misc_feature    1131..1133
                     /label="W43R"
                     /ApEinfo_revcolor="#b4abac"
                     /ApEinfo_fwdcolor="#b4abac"
     RBS             1131..1136
                     /label="Entangled RBS"
```

```
                     /ApEinfo_revcolor="#b4abac"
                     /ApEinfo_fwdcolor="#b4abac"
     CDS             1142..3571
                     /label="Pan-126 Im9"
                     /ApEinfo_revcolor="#ff9ccd"
                     /ApEinfo_fwdcolor="#ff9ccd"

/translation="MPFRYQSAQEGPEPAEQDQGILRRGQRIPYDQGQRTIPRGEPEGSGSDYQPLRQVSADY
QEAGGLQAVQDGPQSYQEQEPPDQGGSAGTGGHQSRDQQRPEQRSQHRLSGYQHHSASGHQPSANSQSVLAER
LRGRGGLFREGHKRADGGRQHRVYREYRSGQQSDGQGRASAGSDQAAAWAGDHVQRGSEEPTPGTQVRQQPGG
SGAAEIHLQRHTRTGGAYTAQRAAPEPHNQGRGVLRGDHLRNGSEEGTGWPYRGTGEGGEQVISDLRGSGTGK
RAGGELPQSQQQGLLRMDHRSTGPEPSGQPRAQGHISNLRTYPVRERSFLRLYAEEQIPPDNRGAEGAGLPAG
PLDRRRSVRPGHLQRGQPGHQPDGARDGVRGETEPLCGIQGPQGAAGGKDREPVQQGGAWQRHPQQPEHGESA
LGRHRGSGLPEGWREEHTELSEHGQHWHPGDLPGRPDRLRRLRDGRTRHQGHHQDDPHQRAGRPGEPGPQPGS
GGERERGAGQSGYERYKAQDQLRHLHERRRRAAERAEQMCWQQEVPSGTGSRLRPGMSWFLLRTAGAEGGRLL
RYHPVRRLRPPIPAGKSSGGAQLFQRGGVQEQDEQTGRGRQAVIHSDPEQPGRISDQEPDRVPGLWQHQPGSA
WYNRLQGDQFQQHQGHHRAVLHQVSAEGQQESGFHGLLRGGASDGEQVSSDQGGSGSDQEDPQPYEYQPQISG
GGSGGGSMELKHSISDYTEAEFLQLVTTICNADTSSEEELVKLVTHFEEMTEHPSGSDLIYYPKEGDDDSPSG
IVNTVKQWRAANGKSGFKQG*"
     CDS             3311..3571
                     /label="Im9"
                     /ApEinfo_revcolor="#ff9ccd"
                     /ApEinfo_fwdcolor="#ff9ccd"

/translation="MELKHSISDYTEAEFLQLVTTICNADTSSEEELVKLVTHFEEMTEHPSGSDLIYYPKEG
DDDSPSGIVNTVKQWRAANGKSGFKQG*"
     terminator      3594..3867
                     /label="Aph 3' Terminator"
                     /ApEinfo_revcolor="#b4abac"
                     /ApEinfo_fwdcolor="#b4abac"
     terminator      complement(4203..4230)
                     /label="T7Te terminator"
                     /ApEinfo_revcolor="#c6c9d1"
                     /ApEinfo_fwdcolor="#c6c9d1"
                     /note="note: phage T7 early transcription
terminator"
     terminator      complement(4246..4317)
                     /label="rrnB T1 terminator"
                     /ApEinfo_revcolor="#c6c9d1"
                     /ApEinfo_fwdcolor="#c6c9d1"
                     /note="note: transcription terminator T1 from the E.
coli rrnB gene"
     CDS             complement(4362..6128)
                     /label="colE9 K12 optimized"
                     /ApEinfo_revcolor="#faac61"
                     /ApEinfo_fwdcolor="#faac61"

/translation="MHHHHHHSGGDGRGHNTGAHSTSGNINGGPTGIGVSGGASDGSGWSSENNPWGGGSGSG
IHWGGGSGRGNGGGNGNSGGGSGTGGNLSAVAAPVAFGFPALSTPGAGGLAVSISASELSAAIAGIIAKLKKV
NLKFTPFGVVLSSLIPSEIAKDDPNMMSKIVTSLPADDITESPVSSLPLDKATVNVNVRVVDDVKDERQNISV
VSGVPMSVPVVDAKPTERPGVFTASIPGAPVLNISVNDSTPAVQTLSPGVTNNTDKDVRPAGFTQGGNTRDAV
IRFPKDSGHNAVYVSVSDVLSPDQVKQRQDEENRRQQEWDATHPVEAAERNYERARAELNQANEDVARNQERQ
AKAVQVYNSRKSELDAANKTLADAIAEIKQFNRFAHDPMAGGHRMWQMAGLKAQRAQTDVNNKQAAFDAAAKE
KSDADAALSAAQERRKQKENKEKDAKDKLDKESKRNKPGKATGKGKPVGDKWLDDAGKDSGAPIPDRIADKLR
```

DKEFKSFDDFRKAVWEEVSKDPELSKNLNPSNKSSVSKGYSPFTPKNQQVGGRKVYELHHDKPISQGGEVYDM
DNIRVTTPKRHIDIHRGK*"
     misc_feature   6136..6149
                     /label="BBa_B0031 ""weak"" RBS 0.07"
                     /ApEinfo_revcolor="#b4abac"
                     /ApEinfo_fwdcolor="#b4abac"
     misc_feature   complement(6171..6205)
                     /label="BBa_j23106"
                     /ApEinfo_revcolor="#c7b0e3"
                     /ApEinfo_fwdcolor="#c7b0e3"
     promoter   6438..6542
                     /label="AmpR promoter"
                     /ApEinfo_revcolor="#c6c9d1"
                     /ApEinfo_fwdcolor="#c6c9d1"
                     /note="gene: bla"
     CDS   6543..7194
                     /label="AmpR"
                     /ApEinfo_revcolor="#993366"
                     /ApEinfo_fwdcolor="#993366"
                     /note="codon_start: 1 transl_table: 1"
     rep_origin   7359..381
                     /label="p15A ori"
                     /ApEinfo_revcolor="#ffef86"
                     /ApEinfo_fwdcolor="#ffef86"
                     /note="direction: RIGHT note: Plasmids containing
the medium-copy-number p15A origin of replication can be propagated in E.
coli cells that contain a second plasmid with the ColE1 origin."
ORIGIN
        1 GGCGCATGAC TTCAAGACTA ACTCCTCTAA ATCAATTACC AGTGGCTGCT
GCCAGTGGTG
       61 CTTTTGCATG TCTTTCCGGG TTGGACTCAA GACGATAGTT ACCGGATAAG
GCGCAGCGGT
      121 CGGACTGAAC GGGGGGTTCG TGCATACAGT CCAGCTTGGA GCGAACTGCC
TACCCGGAAC
      181 TGAGTGTCAG GCGTGGAATG AGACAAACGC GGCCATAACA GCGGAATGAC
ACCGGTAAAC
      241 CGAAAGGCAG GAACAGGAGA GCGCACGAGG GAGCCGCCAG GGGGAAACGC
CTGGTATCTT
      301 TATAGTCCTG TCGGGTTTCG CCACCACTGA TTTGAGCGTC AGATTTCGTG
ATGCTTGTCA
      361 GGGGGGCGGA GCCTATGGAA AAACGGCTTT GCCGCGGCCC TCTCACTTCC
CTGTTAAGTA
      421 TCTTCCTGGC ATCTTCCAGG AAATCTCCGC CCCGTTCGTA AGCCATTTCC
GCTCGCCGCA
      481 GTCGAACGAC CGAGCGTAGC GAGTCAGTGA GCGAGGAAGC GGAATATATC
CTGTATCACA
      541 TATTCTGCTG ACGCACCGGT GCAGCCTTTT TTCTCCTGCC ACATGAAGCA
CTTCACTGAC
      601 ACCCTCATCA GTGCCAACAT AGTAAGCCAG TATACACTCC GCTAGCGCTG
AGGTCTGCCT
      661 CGTGAAGAAG GTGTTGCTGA CTCATACCAG GCCTGAATCG CCCCATCATC
CAGCCAGAAA
      721 GTGAGGGAGC CACGGTTGAT GAGAGCTTTG TTGTAGGTGG ACCAGTTGGT
GATTTTGAAC

781 TTTTGCTTTG CCACGGAACG GTCTGCGTTG TCGGCATGCG CATAATGTGC CTGTCAAATG
841 GACGAAGCAG GGATTCTGCA AACCCTATGC TACTCCGTCA AGCCGTCAAT TGTCTGATTC
901 GTTACCAAct gacagctagc tcagtcctag gtataatgct agcTCCATAC CCGTTTTTTT
961 GGGCTAGAAA TAATTTTGTT TAACTTTAAG AAGGAGATAT ACCCATGGGT TTCAAGCGTA
1021 ATTTCAGTAC ACTTGAGAGC AAGCTTAACC CGAGCTACAT TAGCGGCTTC GTGGACGGTG
1081 AGGGTAGCTT TATGCTGACC ATTATTAAAG ACAATAAATA CAAGCTTGGG AGGAGGGTTG
1141 TATGCCGTTT CGTTATCAGT CTGCACAAGA AGGACCTGAG CCTGCTGAAC AAGATCAAGG
1201 AATTCTTCGA CGTGGGCAAC GTATTCCTTA TGACCAAGGA CAGCGCACAA TACCGCGTGG
1261 AGAGCCTGAA GGGTCTGGAT CTGATTATCA ACCACTTCGA CAAGTATCCG CTGATTACCA
1321 AGAAGCAGGC GGATTACAAG CTGTTCAAGA TGGCCCACAA TCTTATCAAG AACAAGAGCC
1381 ACCTGACCAA GGAGGGTCTG CTGGAACTGG TGGCCATCAA AGCCGTGATC AACAACGGCC
1441 TGAACAACGA TCTCAGCATC GCCTTTCCGG GTATCAACAC CATTCTGCGT CCGGACACCA
1501 GCCTTCCGCA AATTCTCAAT CCGTTCTGGC TGAGCGGCTT CGTGGACGCG GAGGGCTGTT
1561 TCGCGAAGGG CACAAACGTG CTGATGGCGG ACGGCAGCAT AGAGTGTATA GAGAATATAG
1621 AAGTGGGCAA CAAAGTGATG GGCAAGGACG GGCGTCCGCG GGAAGTGATC AAGCTGCCGC
1681 GTGGGCGGGA GACCATGTAC AGCGTGGTTC AGAAGAGCCA ACACCGGGCA CACAAGTCAG
1741 ACAGCAGCCG GGAGGTTCCG GAGCTGCTGA AATTCACCTG CAACGCCACA CACGAACTGG
1801 TGGTGCGTAC ACCGCGCAGC GTGCGGCGCC TGAGCCGCAC AATCAAGGGC GTGGAGTACT
1861 TCGAGGTGAT CACCTTCGAA ATGGGTCAGA AGAAGGCACC GGATGGCCGT ATCGTGGAAC
1921 TGGTGAAGGA GGTGAGCAAG TCATATCCGA TCTCAGAGGG TCCGGAACGG GCAAACGAGC
1981 TGGTGGAGAG TTACCGCAAA GCCAGCAACA AGGCTTACTT CGAATGGACC ATAGAAGCAC
2041 GGGACCTGAG CCTTCTGGGC AGCCACGTGC GCAAGGCCAC ATATCAAACT TACGCACCTA
2101 TCCTGTACGA GAACGATCAT TTCTTCGATT ATATGCAGAA GAGCAAATTC CACCTGACAA
2161 TAGAGGGGCC GAAGGTGCTG GCTTACCTGC CGGGCCTCTG GATCGGAGAC GGTCTGTCAG
2221 ACCGGGCCAC CTTCAGCGTG GACAGCCGGG ACACCAGCCT GATGGAGCGC GTGACGGAGT
2281 ACGCGGAGAA ACTGAACCTC TGTGCGGAAT ACAAGGACCG CAAGGAGCCG CAGGTGGCAA
2341 AGACCGTGAA CCTGTACAGC AAGGTGGTGC GTGGCAACGG CATCCGCAAC AACCTGAACA

2401 CGGAGAATCC GCTCTGGGAC GCCATCGTGG GTCTGGGCTT CCTGAAGGAT GGCGTGAAGA
2461 ACATACCGAG CTTTCTGAGC ACGGACAACA TTGGCACCCG GGAGACCTTC CTGGCCGGCC
2521 TGATAGACTC AGACGGTTAC GTGACGGACG AACACGGCAT CAAGGCCACC ATCAAGACGA
2581 TCCACACCAG CGTGCGGGAC GGCCTGGTGA GCCTGGCCCG CAGCCTGGGT CTGGTGGTGA
2641 GCGTGAACGC GGAGCCGGCC AAAGTGGATA TGAACGGTAC AAAGCACAAG ATCAGTTACG
2701 CCATTTACAT GAGCGGCGGA GACGTGCTGC TGAACGTGCT GAGCAAATGT GCTGGCAGCA
2761 AGAAGTTCCG TCCGGCACCG GCAGCCGCCT TCGCCCGGGA ATGTCGTGGT TTCTACTTCG
2821 AACTGCAGGA GCTGAAGGAG GACGATTATT ACGGTATCAC CCTGTCAGAC GACTCAGACC
2881 ACCAATTCCT GCTGGCAAAT CAAGTGGTGG TGCACAACTG TTTCAGCGTG GTGGTGTTCA
2941 AGAGCAAGAC GAGCAAACTG GGAGAGGCCG TCAAGCTGTC ATTCATTCTG ACCCAGAGCA
3001 ACCGGGACGA ATATCTGATC AAGAGCCTGA TAGAGTACCT GGGCTGTGGC AACACCAGCC
3061 TGGATCCGCG TGGTACAATA GACTTCAAGG TGACCAATTT CAGCAGCATC AAGGACATCA
3121 TCGTGCCGTT CTTCATCAAG TATCCGCTGA AGGGCAACAA GAATCTGGAT TTCACGGACT
3181 TCTGCGAGGT GGTGCGTCTG ATGGAGAACA AGTCTCATCT GACCAAGGAG GGTCTGGATC
3241 AGATCAAGAA GATCCGCAAC CGTATGAATA CCAACCGCAA ATAAGCGGTG GCGGTAGCGG
3301 TGGAGGTAGT ATGGAACTGA AACATAGCAT TAGCGATTAT ACCGAAGCCG AATTTCTGCA
3361 GCTGGTGACC ACCATTTGTA ATGCCGATAC CAGCAGCGAA GAAGAACTGG TGAAACTGGT
3421 GACCCATTTT GAAGAAATGA CCGAACATCC GAGCGGTAGC GATCTGATTT ATTATCCGAA
3481 AGAAGGTGAT GATGATAGCC CGAGCGGTAT TGTGAATACC GTGAAACAGT GGCGTGCCGC
3541 CAATGGTAAA AGCGGTTTTA AACAGGGTTA AGCGGCCGCT CAGAATTGGT TAATTGGTTG
3601 TAACACTGGC AGAGCATTAC GCTGACTTGA CGGGACGGCG GCTTTGTTGA ATAAATCGAA
3661 CTTTTGCTGA GTTGAAGGAT CAGATCACGC ATCTTCCCGA CAACGCAGAC CGTTCCGTGG
3721 CAAAGCAAAA GTTCAAAATC ACCAACTGGT CCACCTACAA CAAAGCTCTC ATCAACCGTG
3781 GCTCCCTCAC TTTCTGGCTG GATGATGGGG CGATTCAGGC CTGGTATGAG TCAGCAACAC
3841 CTTCTTCACG AGGCAGACCT CAGCGCTCAA AGATGCAGGG GTAAAAGCTA ACCGCATCTT
3901 TACCGACAAG GCATCCGGCA GTTCAACAGA TCGGGAAGGG CTGGATTTGC TGAGGATGAA
3961 GGTGGAGGAA GGTGATGTCA TTCTGGTGAA GAAGCTCGAC CGTCTTGGCC GCGACACCGC

4021 CGACATGATC CAACTGATAA AAGAGTTTGA TGCTCAGGGT GTAGCGGTTC GGTTTATTGA
4081 CGACGGGATC AGTACCGACG GTGATATGGG GCAAATGGTG GTCACCATCC TGTCGGCTGT
4141 GGCACAGGCT GAACGCCGGA GGATCCTAGA GCGCACGAAT GAGGGCCGAC AGGAAGCAAA
4201 GCcgcagaaa ggcccacccg aaggtgagcc agtgtgactc tagtagagag cgttcaccga
4261 caaacaacag ataaaacgaa aggcccagtc tttcgactga gcctttcgtt ttatttgatg
4321 cctggctcta gtagcgatct acactagcac tatcagcgtt aTTATTTACC ACGGTGGATG
4381 TCGATGTGGC GTTTGGGAGT CGTGACACGG ATGTTGTCCA TATCGTACAC CTCCCCTCCC
4441 TGCGAAATTG GTTTGTCGTG GTGCAGTTCG TAGACTTTGC GGCCGCCCAC CTGTTGGTTT
4501 TTCGGAGTAA ACGGAGAATA ACCCTTTGAG ACCGAACTCT TGTTGCTGGG ATTTAAGTTT
4561 TTGCTTAATT CCGGATCTTT GCTTACTTCC TCCCATACGG CTTTGCGAAA ATCATCGAAT
4621 GACTTGAACT CCTTGTCACG TAATTTATCT GCAATACGGT CGGGGATTGG AGCGCCACTA
4681 TCCTTGCCTG CGTCATCCAG CCATTTGTCA CCAACGGGTT TGCCTTTCCC GGTCGCTTTT
4741 CCAGGCTTGT TACGCTTAGA CTCCTTGTCC AGCTTGTCTT TAGCGTCTTT CTCCTTATTT
4801 TCTTTTTGTT TGCGGCGTTC TTGTGCTGCT GACAATGCAG CGTCCGCATC TGATTTCTCC
4861 TTGGCTGCAG CATCGAAAGC AGCTTGCTTA TTGTTGACGT CGGTCTGGGC GCGTTGCGCC
4921 TTTAAGCCTG CCATCTGCCA CATGCGGTGG CCGCCAGCCA TTGGATCGTG CGCGAAGCGA
4981 TTGAACTGCT TAATCTCAGC GATGGCGTCT GCCAAAGTTT TATTGGCGGC ATCAAGCTCA
5041 GATTTACGGG AATTATACAC TTGGACGGCT TTCGCTTGGC GTTCTTGATT ACGAGCCACG
5101 TCTTCGTTAG CCTGATTTAA CTCAGCGCGT GCACGTTCAT AGTTACGTTC AGCGGCTTCT
5161 ACGGGGTGCG TAGCATCCCA TTCCTGTTGA CGGCGGTTTT CTTCATCCTG ACGTTGTTTA
5221 ACTTGATCTG GAGACAGCAC ATCTGAAACC GAAACGTACA CCGCGTTATG GCCAGAATCC
5281 TTGGGGAAGC GGATAACCGC ATCGCGTGTG TTACCACCCT GCGTAAACCC AGCTGGACGA
5341 ACATCCTTAT CTGTATTGTT CGTAACTCCG GGCGATAAGG TCTGGACAGC CGGAGTCGAG
5401 TCATTCACTG AAATGTTCAG CACTGGTGCT CCGGGGATGG AGGCTGTGAA TACACCTGGA
5461 CGCTCTGTTG GTTTGGCGTC AACCACGGGG ACACTCATCG GGACCCCGCT AACTACCGAA
5521 ATATTCTGGC GTTCATCCTT TACGTCATCG ACGACACGAA CGTTAACGTT CACTGTCGCC
5581 TTATCAAGCG GCAGAGAGGA AACGGGGGAT TCGGTAATAT CATCCGCCGG CAGACTGGTG

5641 ACAATTTTGC TCATCATGTT GGGATCATCC TTGGCGATTT CTGACGGAAT CAAACTAGAC
5701 AGAACAACGC CGAAGGGCGT AAATTTAAGA TTTACCTTCT TTAACTTCGC GATGATTCCG
5761 GCAATTGCGG CGGACAGCTC GGACGCCGAA ATGGACACCG CCAAGCCACC TGCGCCGGGC
5821 GTAGAAAGCG CTGGGAAACC GAACGCGACA GGCGCTGCTA CTGCGGATAA ATTTCCTCCA
5881 GTCCCGGAGC CGCCTCCACT ATTACCGTTT CCTCCCCCGT TTCCGCGCCC GCTCCCTCCT
5941 CCCCAGTGGA TACCCGATCC GGAGCCCCCA CCCCAAGGGT TGTTCTCGGA AGACCATCCC
6001 GAGCCATCAG ATGCCCCACC GCTGACCCCG ATCCCCGTCG GTCCCCCATT GATATTACCG
6061 CTTGTGCTGT GGGCGCCAGT GTTGTGACCA CGTCCGTCAC CCCCTGAATG GTGGTGGTGA
6121 TGATGCATGG GTATAggttt cctgtgtgac tctagtatgG AATTCtgtga gctagcacta
6181 tacctaggac tgagctagcc gtaaaTGAAA GGAATCAAAT TTGGCCGCAG GCGTACCGTG
6241 GACAGGAACG TCGTGCTGAC GCTTCATCAG AAGGGCACTG GTGCAACGGA AATTGCTCAT
6301 CAGCTCAGTA TTGCCCGCTC CACGGTTTAT AAAATTCTTG AAGACGAAAG GGCCTCGTGA
6361 TACGCCTATT TTTATAGGTT AATGTCATGA TAATAATGGT TTCTTAGACG TCAGGTGGCA
6421 CTTTTCGGGG AAATGTGCGC GGAACCCCTA TTTGTTTATT TTTCTAAATA CATTCAAATA
6481 TGTATCCGCT CATGAGACAA TAACCCTGAT AAATGCTTCA ATAATATTGA AAAAGGAAGA
6541 GTATGAGTAT TCAACATTTC CGTGTCGCCC TTATTCCCTT TTTTGCGGCA TTTTGCCTTC
6601 CTGTTTTTGC TCACCCAGAA ACGCTGGTGA AAGTAAAAGA TGCTGAAGAT CAGTTGGGTG
6661 CACGAGTGGG TTACATCGAA CTGGATCTCA ACAGCGGTAA GATCCTTGAG AGTTTTCGCC
6721 CCGAAGAACG TTTTCCAATG ATGAGCACTT TTAAAGGGAC CGAAGGAGCT AACCGCTTTT
6781 TTGCACAACA TGGGGGATCA TGTAACTCGC CTTGATCGTT GGGAACCGGA GCTGAATGAA
6841 GCCATACCAA ACGACGAGCG TGACACCACG ATGCCTGCAG CAATGGCAAC AACGTTGCGC
6901 AAACTATTAA CTGGCGAACT ACTTACTCTA GCTTCCCGGC AACAATTAAT AGACTGGATG
6961 GAGGCGGATA AAGTTGCAGG ACCACTTCTG CGCTCGGCCC TTCCGGCTGG CTGGTTTATT
7021 GCTGATAAAT CTGGAGCCGG TGAGCGTGGG TCTCGCGGTA TCATTGCAGC ACTGGGGCCA
7081 GATGGTAAGC CCTCCCGTAT CGTAGTTATC TACACGACGG GGAGTCAGGC AACTATGGAT
7141 GAACGAAATA GACAGATCGC TGAGATAGGT GCCTCACTGA TTAAGCATTG GTAACTGTCA
7201 GACCAAGTTT ACTCATATAT ACTTTAGATT GATTTAAAAC TTCATTTTTA ATTTAAAAGG

7261 ATCTAGGTGA AGATCCTTTT TGATAATCTC ATGACCAAAA TCCCTTAACG TGAGTTTTCG
7321 TTCCACTGAG CGTCAGACCC CTTAATAAGA TGATCTTCTT GAGATCGTTT TGGTCTGCGC
7381 GTAATCTCTT GCTCTGAAAA CGAAAAAACC GCCTTGCAGG GCGGTTTTTC GAAGGTTCTC
7441 TGAGCTACCA ACTCTTTGAA CCGAGGTAAC TGGCTTGGAG GAGCGCAGTC ACCAAAACTT
7501 GTCCTTTCAG TTTAGCCTTA ACC
//
