# Supplementary material for: Genetic Entanglement Enables Ultrastable Biocontainment in the Mammalian Gut
Source: ACS Synth Biol. 2025 Sep 7;14(9):3696–708. doi: 10.1021/acssynbio.5c00412 (PMC12455656; doi:10.1021/acssynbio.5c00412)
Supplement: Supplementary file 11 [file sb5c00412_si_011.pdf]

```
LOCUS       pEndo_I-OnuI-533(CmR)   5919 bp ds-DNA     circular     22-
APR-2024
DEFINITION  .
KEYWORDS    "creator:SnapGene License" "marker:AmpR"
FEATURES             Location/Qualifiers
     promoter        909..943
                     /label="J23108 promoter"
                     /ApEinfo_revcolor="#c6c9d1"
                     /ApEinfo_fwdcolor="#c6c9d1"
                     /note="note: bacterial promoter (Registry of
Standard Biological Parts BBa_J23119)"
     RBS             975..997
                     /label="RBS"
                     /ApEinfo_revcolor="#b4abac"
                     /ApEinfo_fwdcolor="#b4abac"
                     /note="note: efficient ribosome binding site from
bacteriophage T7 gene 10 (Olins and Rangwala, 1989)"
     CDS             1005..3284
                     /label="Onu TSM (site 2), ribsored"
                     /ApEinfo_revcolor="#75c6a9"
                     /ApEinfo_fwdcolor="#75c6a9"

/translation="MGSAYMSRRESINPWILTGFADAEGSFLLRIRNNNKSSVGYSTELGFQITLHNKDKSIL
ENIQSTWKVGVIANSGDNAVSLKVTRFEDLKVIIDHFEKYPLITQKLGDYMLFKQAFCVMENKEHLKINGIKE
LVRIKAKLNWGLTDELKKAFPEIISKERSLINKNIPNFKWLAGFTSGEGCFFVNLIKSKSKLGVQVQLVFSIT
QHIKDKNLMNSLITYLGCFAKGTNVLMADGSIECIENIEVGNKVMGKDGRPREVIKLPRGRETMYSVVQKSQH
RAHKSDSSREVPELLKFTCNATHELVVRTPRSVRRLSRTIKGVEYFEVITFEMGQKKAPDGRIVELVKEVSKS
YPISEGPERANELVESYRKASNKAYFEWTIEARDLSLLGSHVRKATYQTYAPILYENDHFFDYMQKSKFHLTI
EGPKVLAYLPGLWIGDGLSDRATFSVDSRDTSLMERVTEYAEKLNLCAEYKDRKEPQVAKTVNLYSKVVRGNG
IRNNLNTENPLWDAIVGLGFLKDGVKNIPSFLSTDNIGTRETFLAGLIDSDGYVTDEHGIKATIKTIHTSVRD
GLVSLARSLGLVVSVNAEPAKVDMNGTKHKISYAIYMSGGDVLLNVLSKCAGSKKFRPAPAAAFARECRGFYF
ELQELKEDDYYGITLSDDSDHQFLLANQVVVHNCGYIKEKNKSEFSWLDFVVTKFSDINDKIIPVFQENTLIG
VKLEDFEDWCKVAKLIEEKKHLTESGLDEIKKIKLNMNKGRVF*"
     RBS             1538..1543
                     /label="RBS"
                     /ApEinfo_revcolor="#b4abac"
                     /ApEinfo_fwdcolor="#b4abac"
     CDS             1547..3970
                     /label="entangled reading frame"
                     /ApEinfo_revcolor="#84b0dc"
                     /ApEinfo_fwdcolor="#84b0dc"

/translation="MLLRESYQEQEQTGRASSTGIQYHTAYQGQESDEQPYHISGLFREGHKRADGGRQHRVY
REYRSGQQSDGQGRASAGSDQAAAWAGDHVQRGSEEPTPGTQVRQQPGGSGAAEIHLQRHTRTGGAYTAQRAA
PEPHNQGRGVLRGDHLRNGSEEGTGWPYRGTGEGGEQVISDLRGSGTGKRAGGELPQSQQQGLLRMDHRSTGP
EPSGQPRAQGHISNLRTYPVRERSFLRLYAEEQIPPDNRGTEGAGLPAGPLDRRRSVRPGHLQRGQPGHQPDG
ARDGVRGETEPLCGIQGPQGAAGGKDREPVQQGGAWQRHPQQPEHGESALGRHRGSGLPEGWREEHTELSEHG
QHWHPGDLPGRPDRLRRLRDGRTRHQGHHQDDPHQRAGRPGEPGPQPGSGGERERGAGQSGYERYKAQDQLRH
LHERRRRAAERAEQMCWQQEVPSGTGSRLRPGMSWFLLRTAGAEGGRLLRYHPVRRLRPPIPAGKSSGGAQLW
LYQGEEQVRIQLAGLRGDQVLRYQRQDHTSVPGEYPDWRQAGGFRGLVQSGQTYRGEETSYGERSGRNQEDQT
QYEQGPGVLSGGGSGGGSMEKKITGYTTVDISQWHRKEHFEAFQSVAQCTYNQTVQLDITAFLKTVKKNKHKF
YPAFIHILARLMNAHPEFRMAMKDGELVIWDSVHPCYTVFHEQTETFSSLWSEYHDDFRQFLHIYSQDVACYG
ENLAYFPKGFIENMFFVSANPWVSFTSFDLNVANMDNFFAPVFTMGKYYTQGDKVLMPLAIQVHHAVCDGFHV
GRMLNELQQYCDEWQGGA*"
```

```
     CDS             3285..3310
                     /label="GS linker"
                     /ApEinfo_revcolor="#993366"
                     /ApEinfo_fwdcolor="#993366"
                     /note="codon_start: 3 transl_table: 1"
     CDS             3311..3970
                     /label="CmR"
                     /ApEinfo_revcolor="#85dae9"
                     /ApEinfo_fwdcolor="#85dae9"

/translation="MEKKITGYTTVDISQWHRKEHFEAFQSVAQCTYNQTVQLDITAFLKTVKKNKHKFYPAF
IHILARLMNAHPEFRMAMKDGELVIWDSVHPCYTVFHEQTETFSSLWSEYHDDFRQFLHIYSQDVACYGENLA
YFPKGFIENMFFVSANPWVSFTSFDLNVANMDNFFAPVFTMGKYYTQGDKVLMPLAIQVHHAVCDGFHVGRML
NELQQYCDEWQGGA*"
     terminator      3993..4266
                     /label="Aph 3' Terminator"
                     /ApEinfo_revcolor="#b4abac"
                     /ApEinfo_fwdcolor="#b4abac"
     promoter        4834..4938
                     /label="AmpR promoter"
                     /ApEinfo_revcolor="#c6c9d1"
                     /ApEinfo_fwdcolor="#c6c9d1"
                     /note="gene: bla"
     CDS             4939..5590
                     /label="AmpR"
                     /ApEinfo_revcolor="#993366"
                     /ApEinfo_fwdcolor="#993366"
                     /note="codon_start: 1 transl_table: 1"
     rep_origin      5755..381
                     /label="p15A ori"
                     /ApEinfo_revcolor="#ffef86"
                     /ApEinfo_fwdcolor="#ffef86"
                     /note="direction: RIGHT note: Plasmids containing
the medium-copy-number p15A origin of replication can be propagated in E.
coli cells that contain a second plasmid with the ColE1 origin."
ORIGIN
        1 GGCGCATGAC TTCAAGACTA ACTCCTCTAA ATCAATTACC AGTGGCTGCT
GCCAGTGGTG
       61 CTTTTGCATG TCTTTCCGGG TTGGACTCAA GACGATAGTT ACCGGATAAG
GCGCAGCGGT
      121 CGGACTGAAC GGGGGGTTCG TGCATACAGT CCAGCTTGGA GCGAACTGCC
TACCCGGAAC
      181 TGAGTGTCAG GCGTGGAATG AGACAAACGC GGCCATAACA GCGGAATGAC
ACCGGTAAAC
      241 CGAAAGGCAG GAACAGGAGA GCGCACGAGG GAGCCGCCAG GGGGAAACGC
CTGGTATCTT
      301 TATAGTCCTG TCGGGTTTCG CCACCACTGA TTTGAGCGTC AGATTTCGTG
ATGCTTGTCA
      361 GGGGGGCGGA GCCTATGGAA AAACGGCTTT GCCGCGGCCC TCTCACTTCC
CTGTTAAGTA
      421 TCTTCCTGGC ATCTTCCAGG AAATCTCCGC CCCGTTCGTA AGCCATTTCC
GCTCGCCGCA
      481 GTCGAACGAC CGAGCGTAGC GAGTCAGTGA GCGAGGAAGC GGAATATATC
CTGTATCACA
```

541 TATTCTGCTG ACGCACCGGT GCAGCCTTTT TTCTCCTGCC ACATGAAGCA CTTCACTGAC
601 ACCCTCATCA GTGCCAACAT AGTAAGCCAG TATACACTCC GCTAGCGCTG AGGTCTGCCT
661 CGTGAAGAAG GTGTTGCTGA CTCATACCAG GCCTGAATCG CCCCATCATC CAGCCAGAAA
721 GTGAGGGAGC CACGGTTGAT GAGAGCTTTG TTGTAGGTGG ACCAGTTGGT GATTTTGAAC
781 TTTTGCTTTG CCACGGAACG GTCTGCGTTG TCGGCATGCG CATAATGTGC CTGTCAAATG
841 GACGAAGCAG GGATTCTGCA AACCCTATGC TACTCCGTCA AGCCGTCAAT TGTCTGATTC
901 GTTACCAAct gacagctagc tcagtcctag gtataatgct agcTCCATAC CCGTTTTTTT
961 GGGCTAGAAA TAATTTTGTT TAACTTTAAG AAGGAGATAT ACCCATGGGT AGCGCATATA
1021 TGAGCCGCCG TGAAAGCATC AATCCCTGGA TCCTGACAGG CTTTGCAGAT GCCGAGGGTA
1081 GCTTCCTGCT TCGTATTCGT AATAATAATA AGAGCAGTGT GGGTTATAGC ACCGAACTGG
1141 GCTTCCAGAT CACCCTGCAC AATAAAGATA AGTCTATCCT GGAGAACATT CAAAGCACCT
1201 GGAAAGTTGG TGTTATTGCA AATAGTGGCG ATAATGCAGT TAGCCTTAAA GTGACCCGCT
1261 TCGAAGATCT TAAAGTGATC ATTGATCACT TCGAGAAGTA TCCGCTTATC ACCCAGAAGC
1321 TGGGCGATTA TATGCTCTTC AAACAAGCCT TCTGTGTGAT GGAGAACAAA GAACACCTGA
1381 AGATCAACGG CATTAAAGAA CTTGTTCGTA TCAAAGCCAA ACTGAACTGG GGTCTTACCG
1441 ACGAACTTAA GAAGGCATTC CCAGAAATTA TTAGCAAAGA ACGTAGCCTG ATTAATAAGA
1501 ACATTCCGAA CTTTAAATGG CTTGCCGGCT TCACCTCAGG AGaGGGATGC TTCTTCGTGA
1561 ATCTTATCAA GAGCAAGAGC AAACTGGGCG TGCAAGTTCA ACTGGTATTC AGTATCACAC
1621 AGCATATCAA GGACAAGAAT CTGATGAACA GCCTTATCAC ATATCTGGGC TGTTTCGCGA
1681 AGGGCACAAA CGTGCTGATG GCGGACGGCA GCATAGAGTG TATAGAGAAT ATAGAAGTGG
1741 GCAACAAAGT GATGGGCAAG GACGGGCGTC CGCGGGAAGT GATCAAGCTG CCGCGTGGGC
1801 GGGAGACCAT GTACAGCGTG GTTCAGAAGA GCCAACACCG GGCACACAAG TCAGACAGCA
1861 GCCGGGAGGT TCCGGAGCTG CTGAAATTCA CCTGCAACGC CACACACGAA CTGGTGGTGC
1921 GTACACCGCG CAGCGTGCGG CGCCTGAGCC GCACAATCAA GGGCGTGGAG TACTTCGAGG
1981 TGATCACCTT CGAAATGGGT CAGAAGAAGG CACCGGATGG CCGTATCGTG GAACTGGTGA
2041 AGGAGGTGAG CAAGTCATAT CCGATCTCAG AGGGTCCGGA ACGGGCAAAC GAGCTGGTGG
2101 AGAGTTACCG CAAAGCCAGC AACAAGGCTT ACTTCGAATG GACCATAGAA GCACGGGACC

2161 TGAGCCTTCT GGGCAGCCAC GTGCGCAAGG CCACATATCA AACTTACGCA CCTATCCTGT
2221 ACGAGAACGA TCATTTCTTC GATTATATGC AGAAGAGCAA ATTCCACCTG ACAATAGAGG
2281 GACCGAAGGT GCTGGCTTAC CTGCCGGGCC TCTGGATCGG AGACGGTCTG TCAGACCGGG
2341 CCACCTTCAG CGTGGACAGC CGGGACACCA GCCTGATGGA GCGCGTGACG GAGTACGCGG
2401 AGAAACTGAA CCTCTGTGCG GAATACAAGG ACCGCAAGGA GCCGCAGGTG GCAAAGACCG
2461 TGAACCTGTA CAGCAAGGTG GTGCGTGGCA ACGGCATCCG CAACAACCTG AACACGGAGA
2521 ATCCGCTCTG GGACGCCATC GTGGGTCTGG GCTTCCTGAA GGATGGCGTG AAGAACATAC
2581 CGAGCTTTCT GAGCACGGAC AACATTGGCA CCCGGGAGAC CTTCCTGGCC GGCCTGATAG
2641 ACTCAGACGG TTACGTGACG GACGAACACG GCATCAAGGC CACCATCAAG ACGATCCACA
2701 CCAGCGTGCG GGACGGCCTG GTGAGCCTGG CCCGCAGCCT GGGTCTGGTG GTGAGCGTGA
2761 ACGCGGAGCC GGCCAAAGTG GATATGAACG GTACAAAGCA CAAGATCAGT TACGCCATTT
2821 ACATGAGCGG CGGAGACGTG CTGCTGAACG TGCTGAGCAA ATGTGCTGGC AGCAAGAAGT
2881 TCCGTCCGGC ACCGGCAGCC GCCTTCGCCC GGGAATGTCG TGGTTTCTAC TTCGAACTGC
2941 AGGAGCTGAA GGAGGACGAT TATTACGGTA TCACCCTGTC AGACGACTCA GACCACCAAT
3001 TCCTGCTGGC AAATCAAGTG GTGGTGCACA ACTGTGGTTA TATCAAGGAG AAGAACAAGT
3061 CAGAATTCAG CTGGCTGGAC TTCGTGGTGA CCAAGTTCTC AGATATCAAC GACAAGATCA
3121 TACCAGTGTT CCAGGAGAAT ACCCTGATTG GCGTCAAGCT GGAGGATTTC GAGGACTGGT
3181 GCAAAGTGGC CAAACTTATA GAGGAGAAGA AACATCTTAC GGAGAGCGGT CTGGACGAAA
3241 TCAAGAAGAT CAAACTCAAT ATGAACAAGG GCCGGGTGTT CTGAGCGGTG GCGGTAGCGG
3301 TGGAGGTAGT ATGGAGAAAA AAATCACTGG ATATACCACC GTTGATATAT CCCAATGGCA
3361 TCGTAAAGAA CATTTTGAGG CATTTCAGTC AGTTGCTCAA TGTACCTATA ACCAGACCGT
3421 TCAGCTGGAT ATTACGGCCT TTTTAAAGAC CGTAAAGAAA AATAAGCACA AGTTTTATCC
3481 GGCCTTTATT CACATTCTTG CCCGCCTGAT GAATGCTCAT CCGGAGTTCC GTATGGCAAT
3541 GAAAGACGGT GAGCTGGTGA TATGGGATAG TGTTCACCCT TGTTACACCG TTTTCCATGA
3601 GCAAACTGAA ACGTTTTCAT CGCTCTGGAG TGAATACCAC GACGATTTCC GGCAGTTTCT
3661 ACACATATAT TCGCAAGATG TGGCGTGTTA CGGTGAAAAC CTGGCCTATT TCCCTAAAGG
3721 GTTTATTGAG AATATGTTTT TCGTCTCAGC CAATCCCTGG GTGAGTTTCA CCAGTTTTGA

3781 TTTAAACGTG GCCAATATGG ACAACTTCTT CGCCCCCGTT TTCACTATGG GCAAATATTA
3841 TACGCAAGGC GACAAGGTGC TGATGCCGCT GGCGATTCAG GTTCATCATG CCGTCTGTGA
3901 TGGCTTCCAT GTCGGCAGAA TGCTTAATGA ATTACAACAG TACTGCGATG AGTGGCAGGG
3961 CGGGGCGTAA GCGGCCGCTC AGAATTGGTT AATTGGTTGT AACACTGGCA GAGCATTACG
4021 CTGACTTGAC GGGACGGCGG CTTTGTTGAA TAAATCGAAC TTTTGCTGAG TTGAAGGATC
4081 AGATCACGCA TCTTCCCGAC AACGCAGACC GTTCCGTGGC AAAGCAAAAG TTCAAAATCA
4141 CCAACTGGTC CACCTACAAC AAAGCTCTCA TCAACCGTGG CTCCCTCACT TTCTGGCTGG
4201 ATGATGGGGC GATTCAGGCC TGGTATGAGT CAGCAACACC TTCTTCACGA GGCAGACCTC
4261 AGCGCTCAAA GATGCAGGGG TAAAAGCTAA CCGCATCTTT ACCGACAAGG CATCCGGCAG
4321 TTCAACAGAT CGGGAAGGGC TGGATTTGCT GAGGATGAAG GTGGAGGAAG GTGATGTCAT
4381 TCTGGTGAAG AAGCTCGACC GTCTTGGCCG CGACACCGCC GACATGATCC AACTGATAAA
4441 AGAGTTTGAT GCTCAGGGTG TAGCGGTTCG GTTTATTGAC GACGGGATCA GTACCGACGG
4501 TGATATGGGG CAAATGGTGG TCACCATCCT GTCGGCTGTG GCACAGGCTG AACGCCGGAG
4561 GATCCTAGAG CGCACGAATG AGGGCCGACA GGAAGCAAAG CTGAAAGGAA TCAAATTTGG
4621 CCGCAGGCGT ACCGTGGACA GGAACGTCGT GCTGACGCTT CATCAGAAGG GCACTGGTGC
4681 AACGGAAATT GCTCATCAGC TCAGTATTGC CCGCTCCACG GTTTATAAAA TTCTTGAAGA
4741 CGAAAGGGCC TCGTGATACG CCTATTTTTA TAGGTTAATG TCATGATAAT AATGGTTTCT
4801 TAGACGTCAG GTGGCACTTT TCGGGGAAAT GTGCGCGGAA CCCCTATTTG TTTATTTTTC
4861 TAAATACATT CAAATATGTA TCCGCTCATG AGACAATAAC CCTGATAAAT GCTTCAATAA
4921 TATTGAAAAA GGAAGAGTAT GAGTATTCAA CATTTCCGTG TCGCCCTTAT TCCCTTTTTT
4981 GCGGCATTTT GCCTTCCTGT TTTTGCTCAC CCAGAAACGC TGGTGAAAGT AAAAGATGCT
5041 GAAGATCAGT TGGGTGCACG AGTGGGTTAC ATCGAACTGG ATCTCAACAG CGGTAAGATC
5101 CTTGAGAGTT TTCGCCCCGA AGAACGTTTT CCAATGATGA GCACTTTTAA AGGGACCGAA
5161 GGAGCTAACC GCTTTTTTGC ACAACATGGG GGATCATGTA ACTCGCCTTG ATCGTTGGGA
5221 ACCGGAGCTG AATGAAGCCA TACCAAACGA CGAGCGTGAC ACCACGATGC CTGCAGCAAT
5281 GGCAACAACG TTGCGCAAAC TATTAACTGG CGAACTACTT ACTCTAGCTT CCCGGCAACA
5341 ATTAATAGAC TGGATGGAGG CGGATAAAGT TGCAGGACCA CTTCTGCGCT CGGCCCTTCC

5401 GGCTGGCTGG TTTATTGCTG ATAAATCTGG AGCCGGTGAG CGTGGGTCTC GCGGTATCAT
5461 TGCAGCACTG GGGCCAGATG GTAAGCCCTC CCGTATCGTA GTTATCTACA CGACGGGGAG
5521 TCAGGCAACT ATGGATGAAC GAAATAGACA GATCGCTGAG ATAGGTGCCT CACTGATTAA
5581 GCATTGGTAA CTGTCAGACC AAGTTTACTC ATATATACTT TAGATTGATT TAAAACTTCA
5641 TTTTTAATTT AAAAGGATCT AGGTGAAGAT CCTTTTTGAT AATCTCATGA CCAAAATCCC
5701 TTAACGTGAG TTTTCGTTCC ACTGAGCGTC AGACCCCTTA ATAAGATGAT CTTCTTGAGA
5761 TCGTTTTGGT CTGCGCGTAA TCTCTTGCTC TGAAAACGAA AAAACCGCCT TGCAGGGCGG
5821 TTTTTCGAAG GTTCTCTGAG CTACCAACTC TTTGAACCGA GGTAACTGGC TTGGAGGAGC
5881 GCAGTCACCA AAACTTGTCC TTTCAGTTTA GCCTTAACC
//
